# Supplementary figures and images for: High genome plasticity and frequent genetic exchange in Leishmania tropica isolates from Afghanistan, Iran and Syria
Source: PLoS Negl Trop Dis. 2021 Dec 30;15(12):e0010110. doi: 10.1371/journal.pntd.0010110 (PMC8754299; doi:10.1371/journal.pntd.0010110)

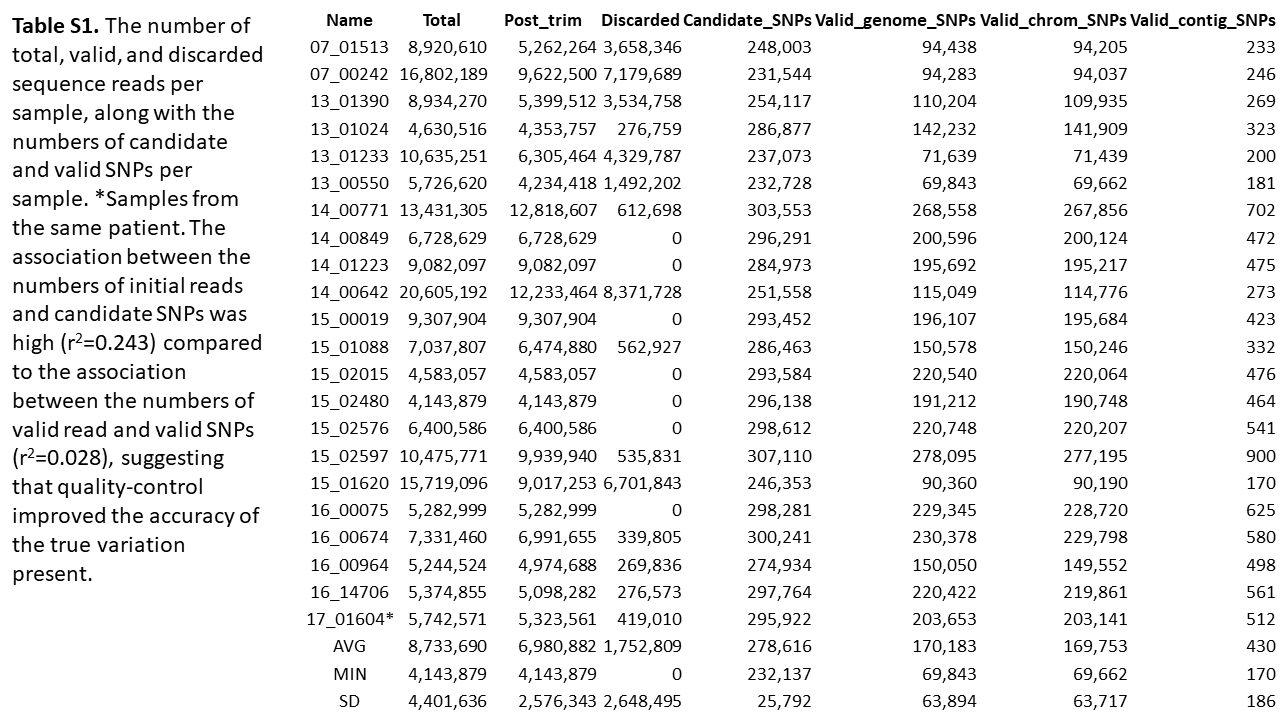

Supplement: S1 Table — (TIF) [file pntd.0010110.s001.tif]

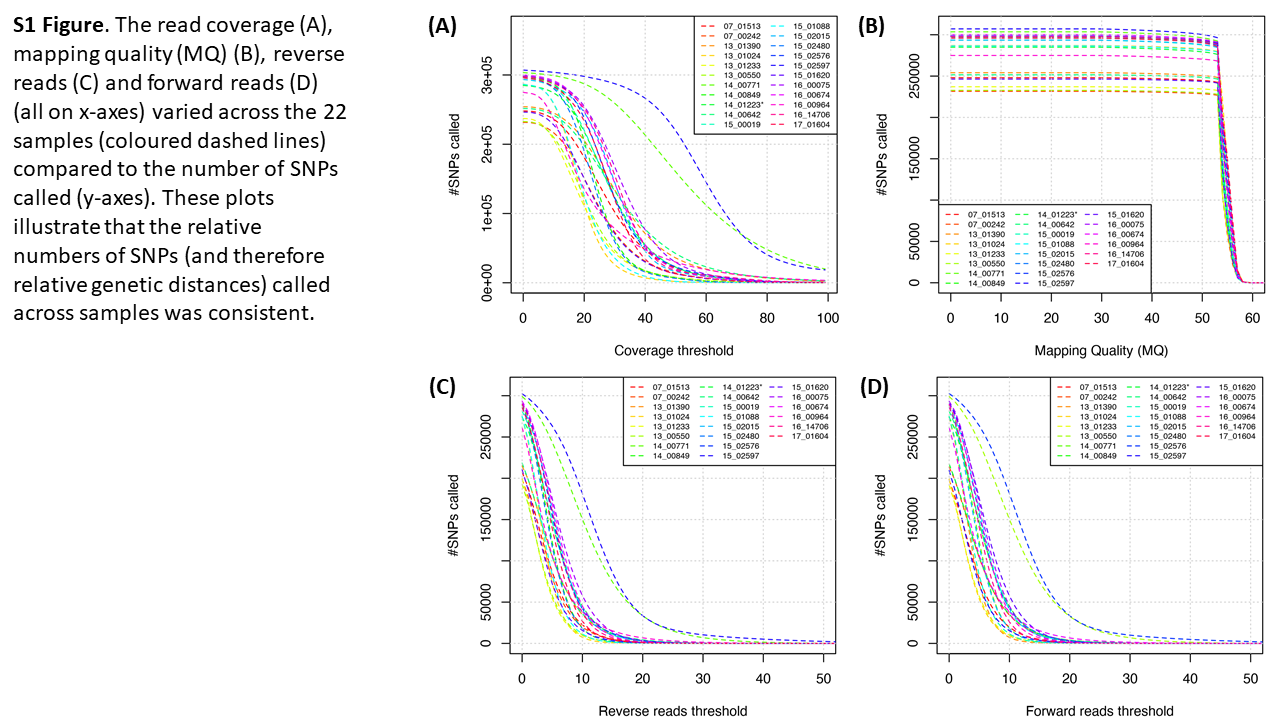

Supplement: S1 Fig — The read coverage (A), mapping quality (MQ) (B), reverse reads (C) and forward reads (D) (all on x-axes) varied across the 22 samples (coloured dashed lines) compared to the number of SNPs called (y-axes). (TIF) [file pntd.0010110.s002.tif]

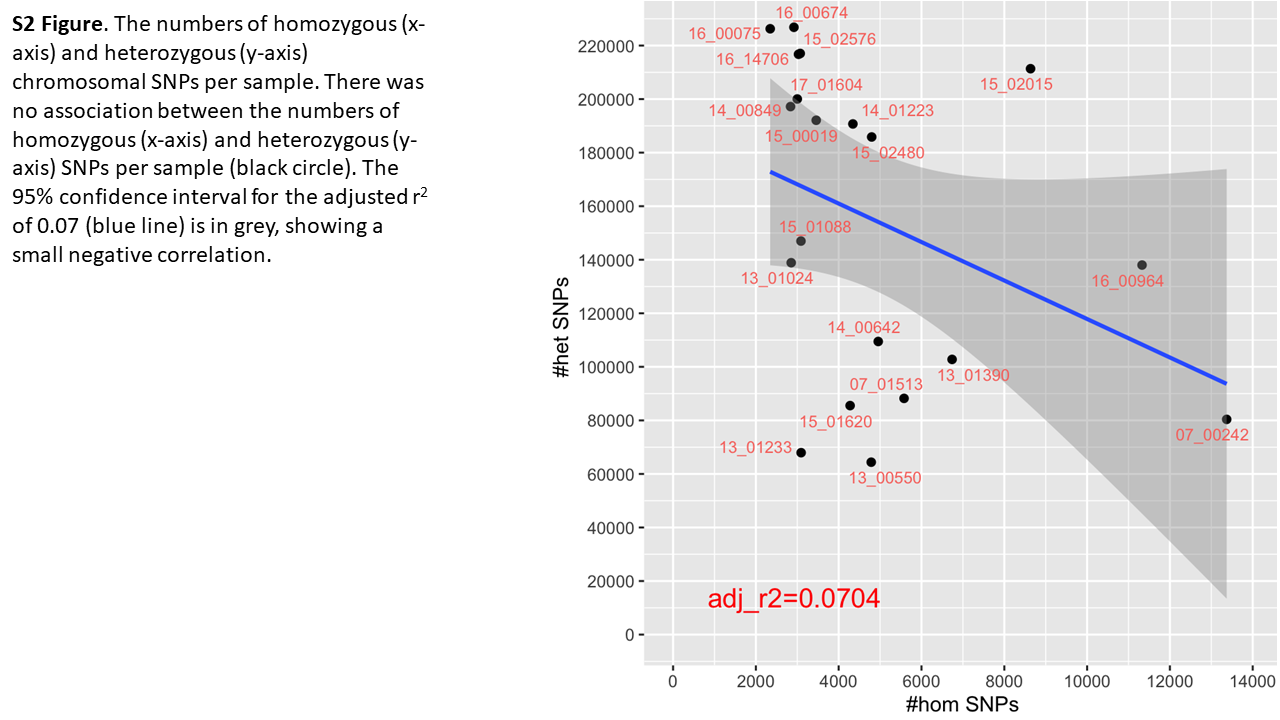

Supplement: S2 Fig — (TIF) [file pntd.0010110.s003.tif]

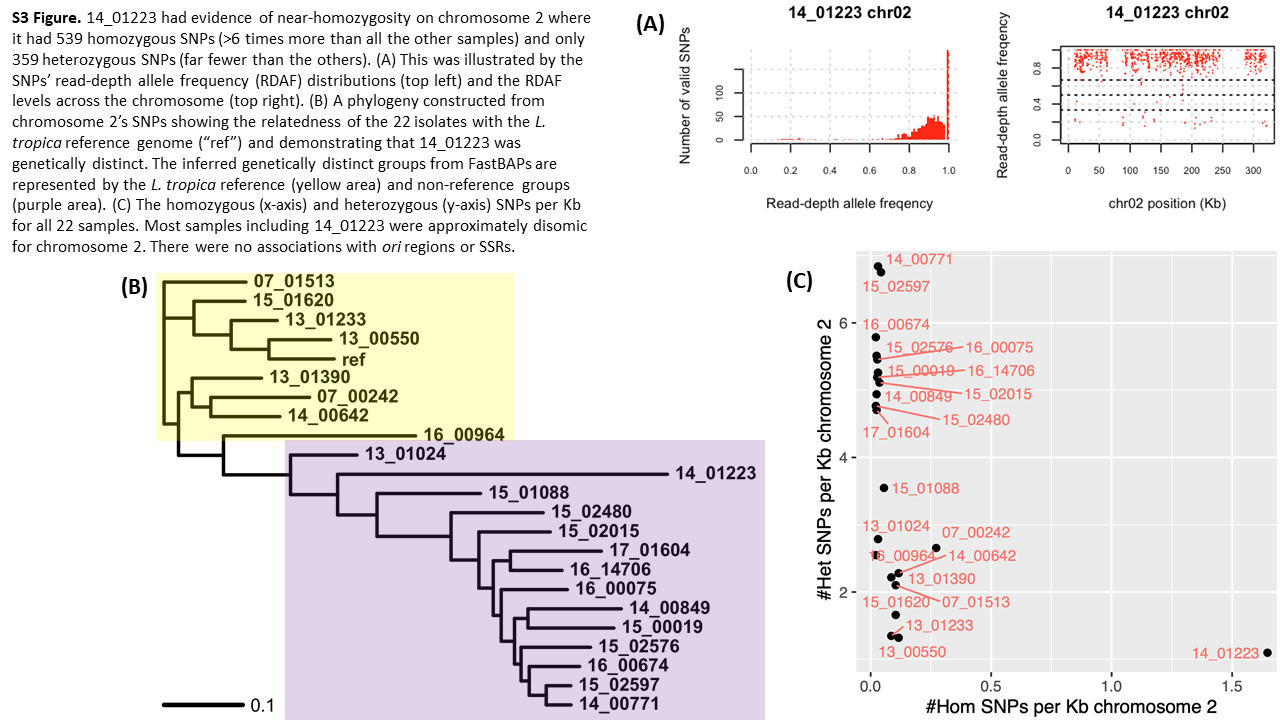

Supplement: S3 Fig — (TIF) [file pntd.0010110.s004.tif]

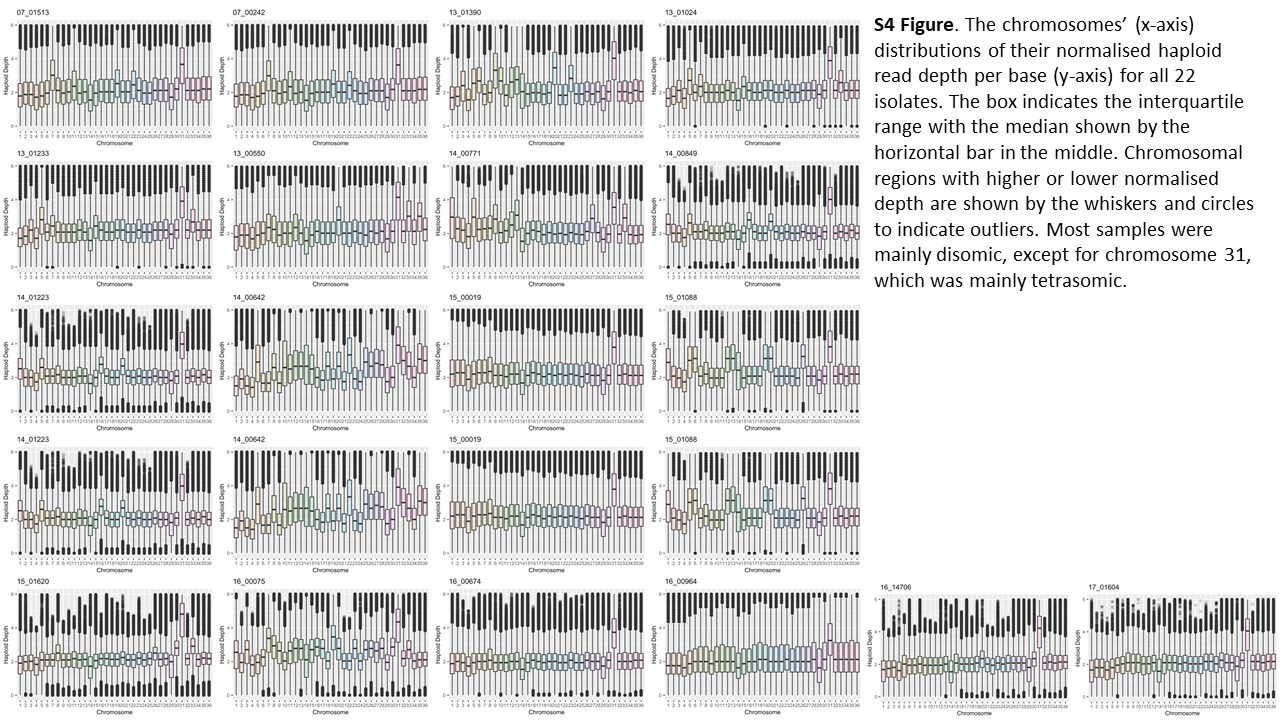

Supplement: S4 Fig — (TIF) [file pntd.0010110.s005.tif]

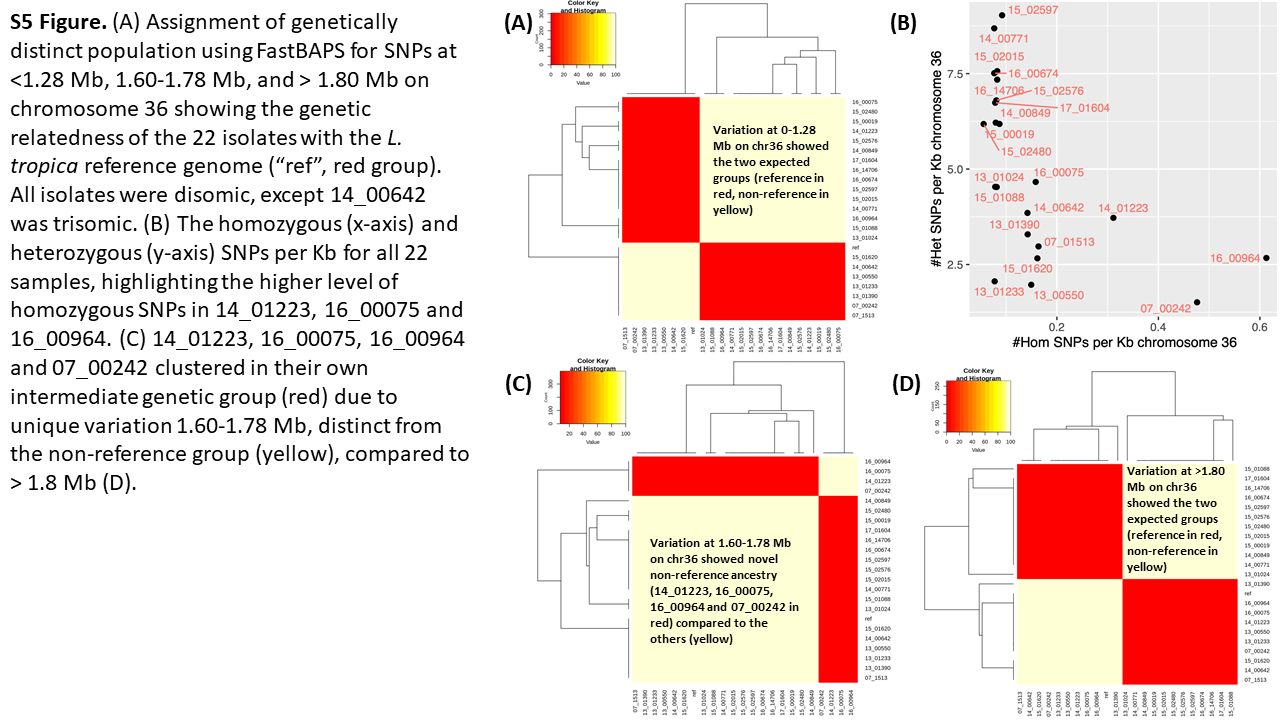

Supplement: S5 Fig — (A) Assignment of genetically distinct population using FastBAPS for SNPs at <1.28 Mb, 1.60–1.78 Mb, and > 1.80 Mb on chromosome 36 showing the genetic relatedness of the 22 isolates with the L. tropica reference genome (“ref”, red group). (TIF) [file pntd.0010110.s006.tif]

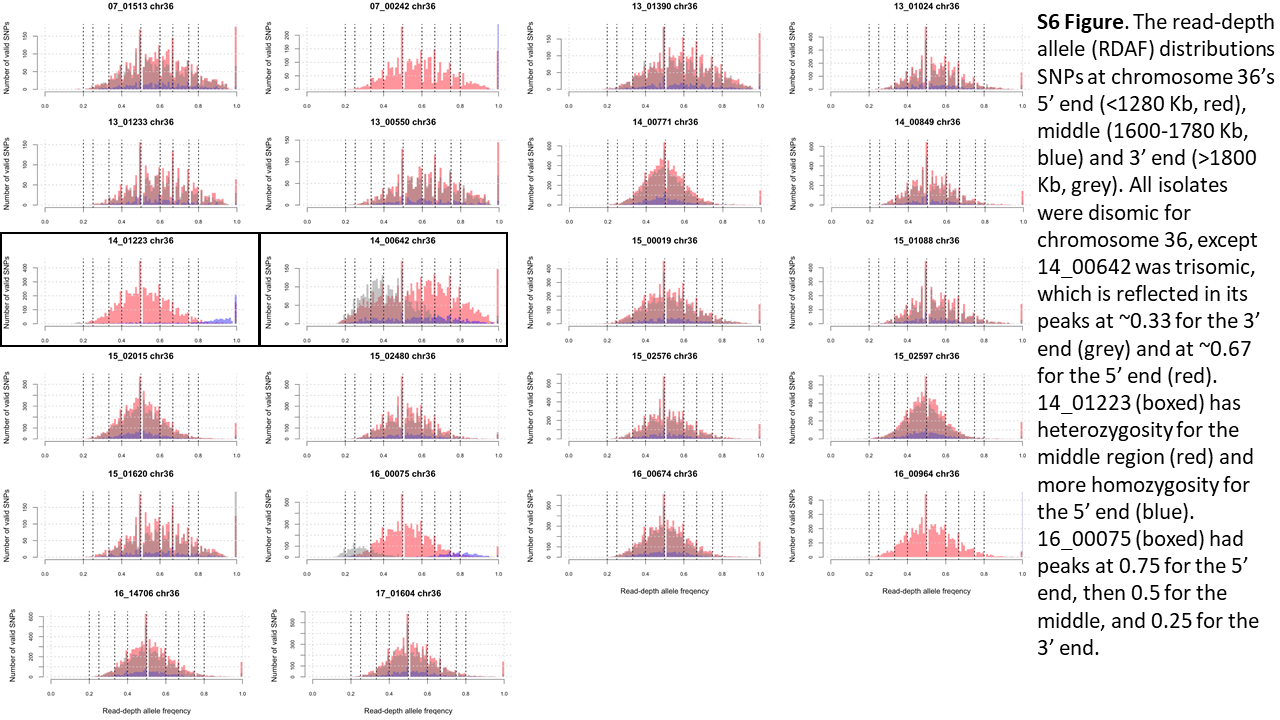

Supplement: S6 Fig — (TIF) [file pntd.0010110.s007.tif]

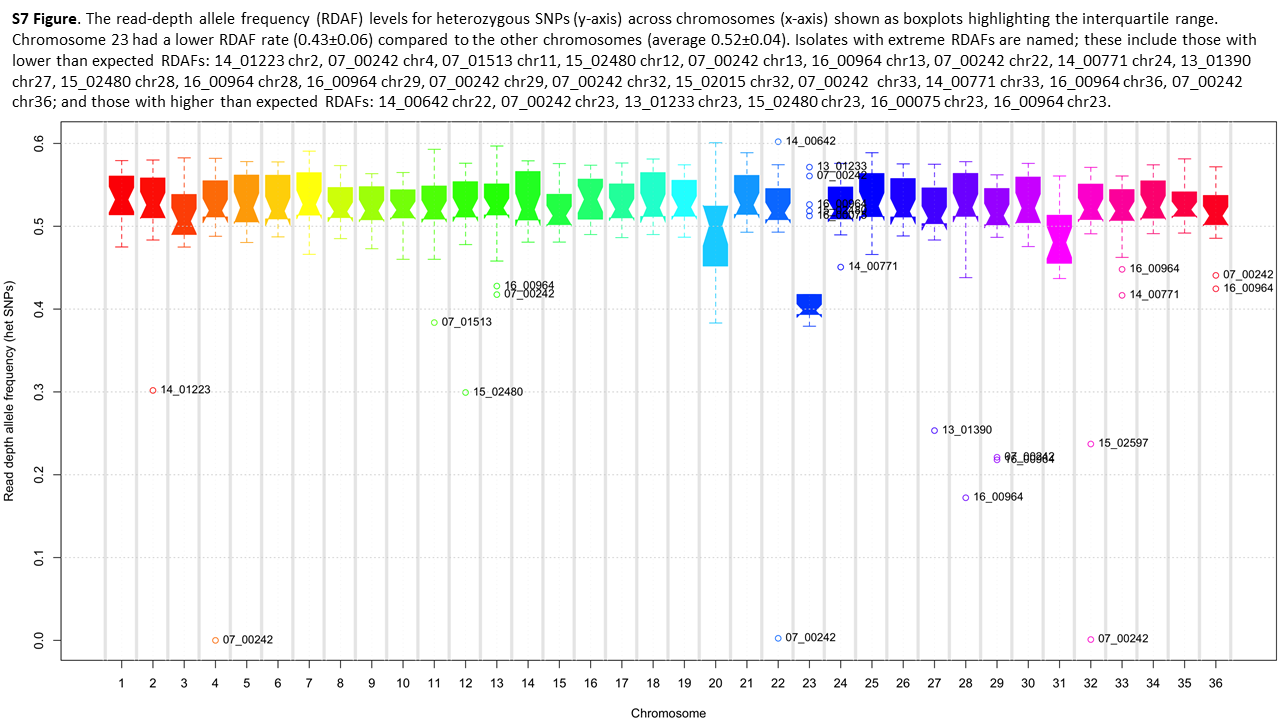

Supplement: S7 Fig — (TIF) [file pntd.0010110.s008.tif]

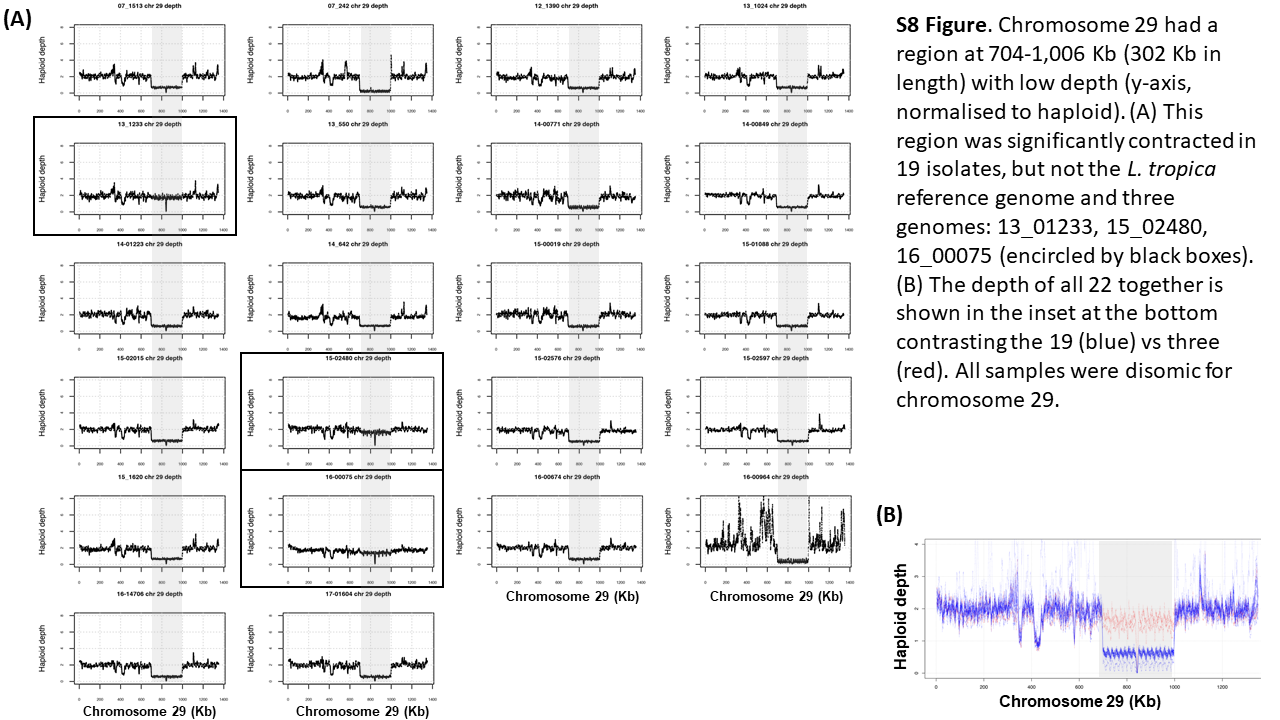

Supplement: S8 Fig — (TIF) [file pntd.0010110.s009.tif]

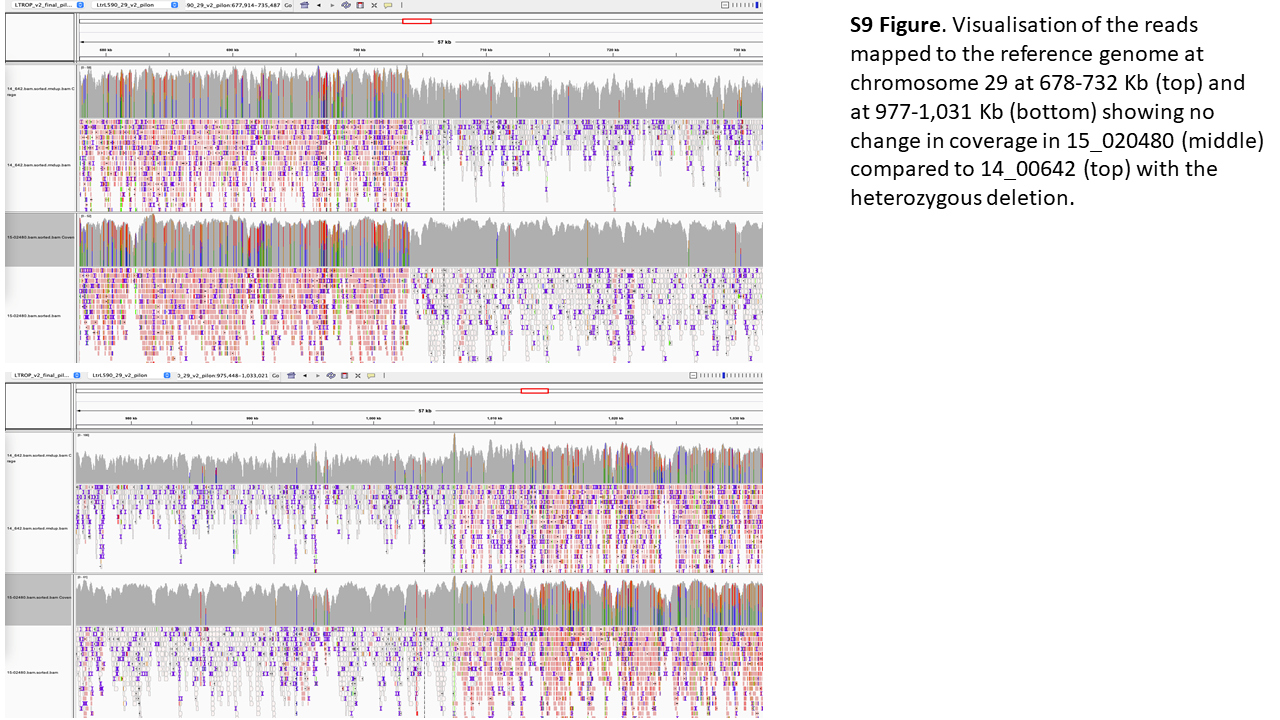

Supplement: S9 Fig — (TIF) [file pntd.0010110.s010.tif]

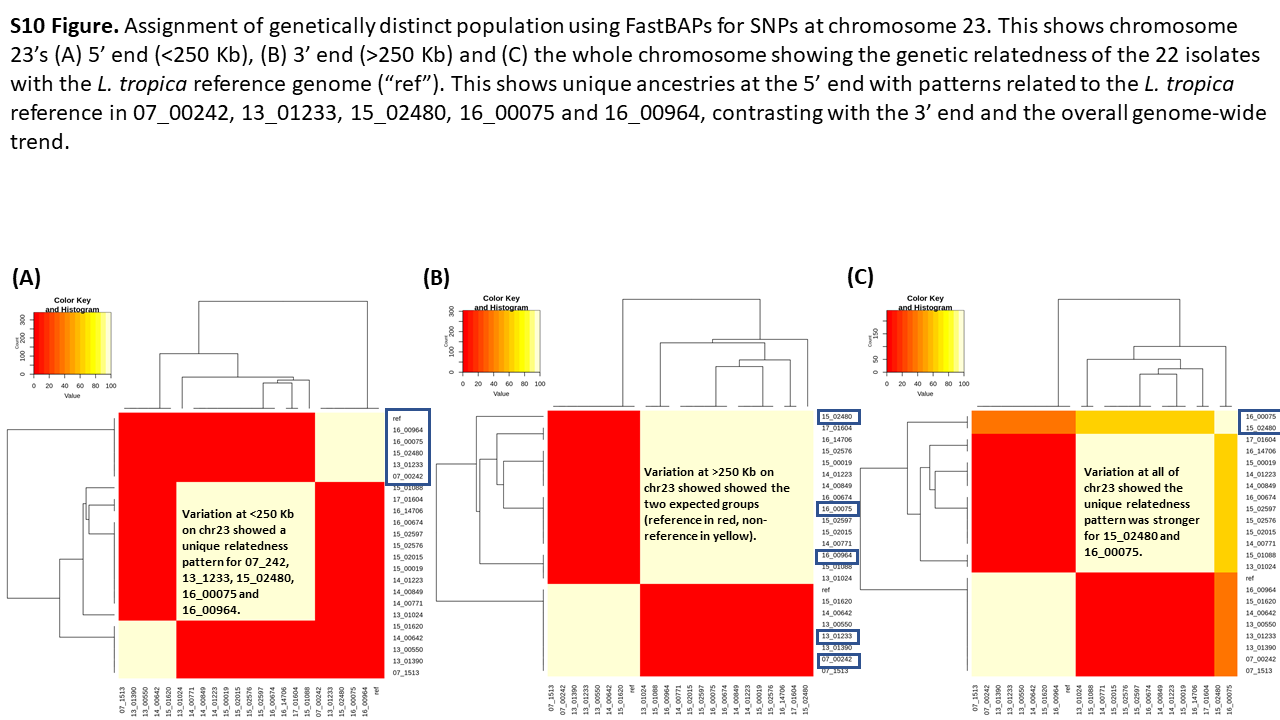

Supplement: S10 Fig — (TIF) [file pntd.0010110.s011.tif]

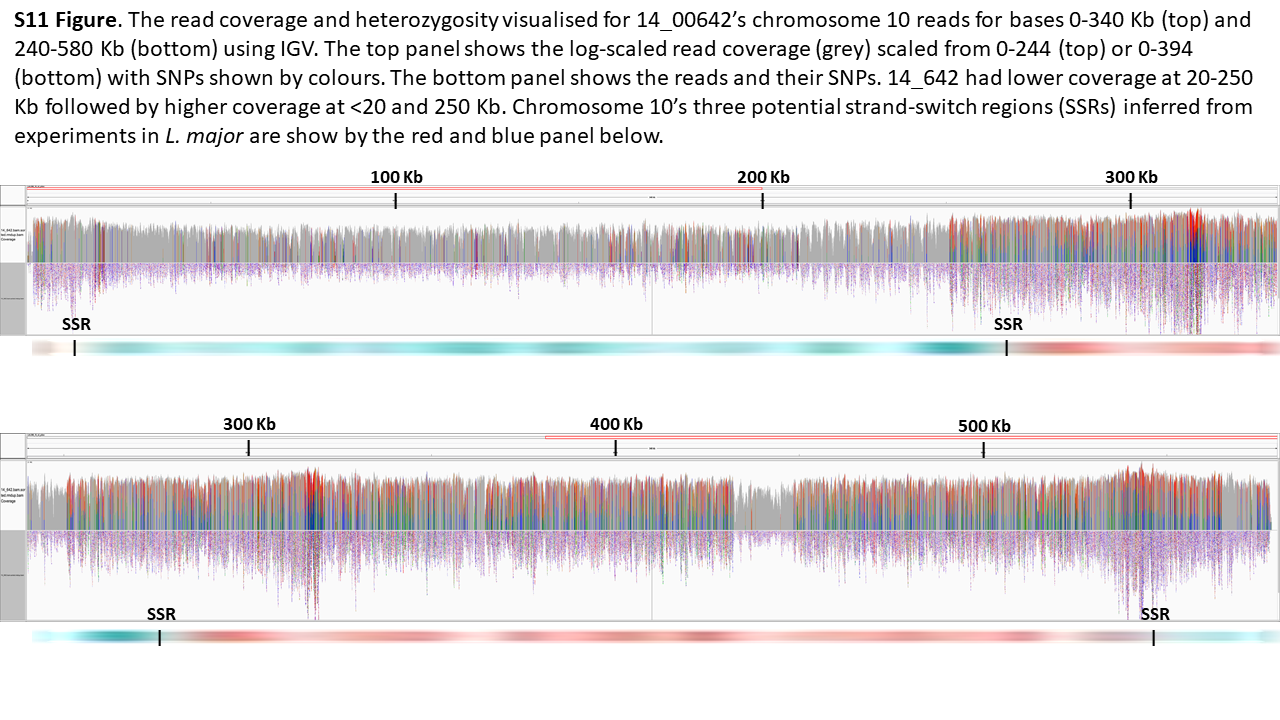

Supplement: S11 Fig — (TIF) [file pntd.0010110.s012.tif]

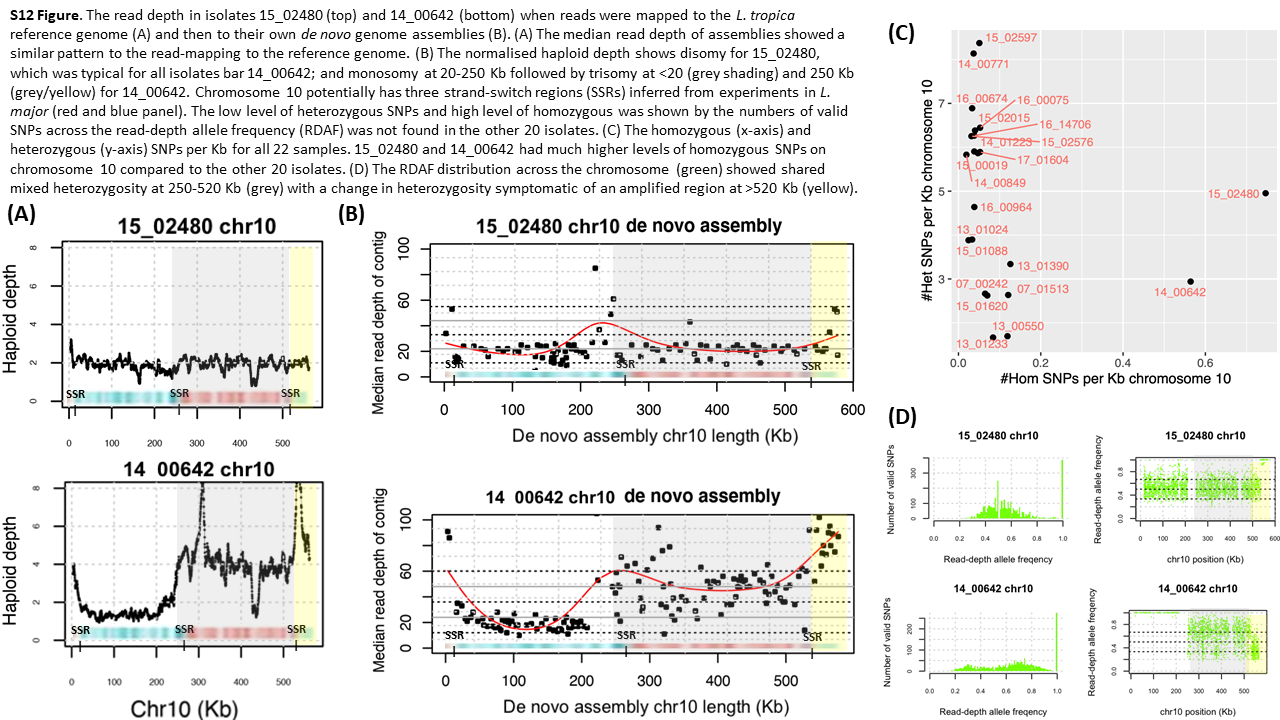

Supplement: S12 Fig — The read depth in isolates 15_02480 (top) and 14_00642 (bottom) when reads were mapped to the L. tropica reference genome (A) and then to their own de novo genome assemblies (B). (TIF) [file pntd.0010110.s013.tif]

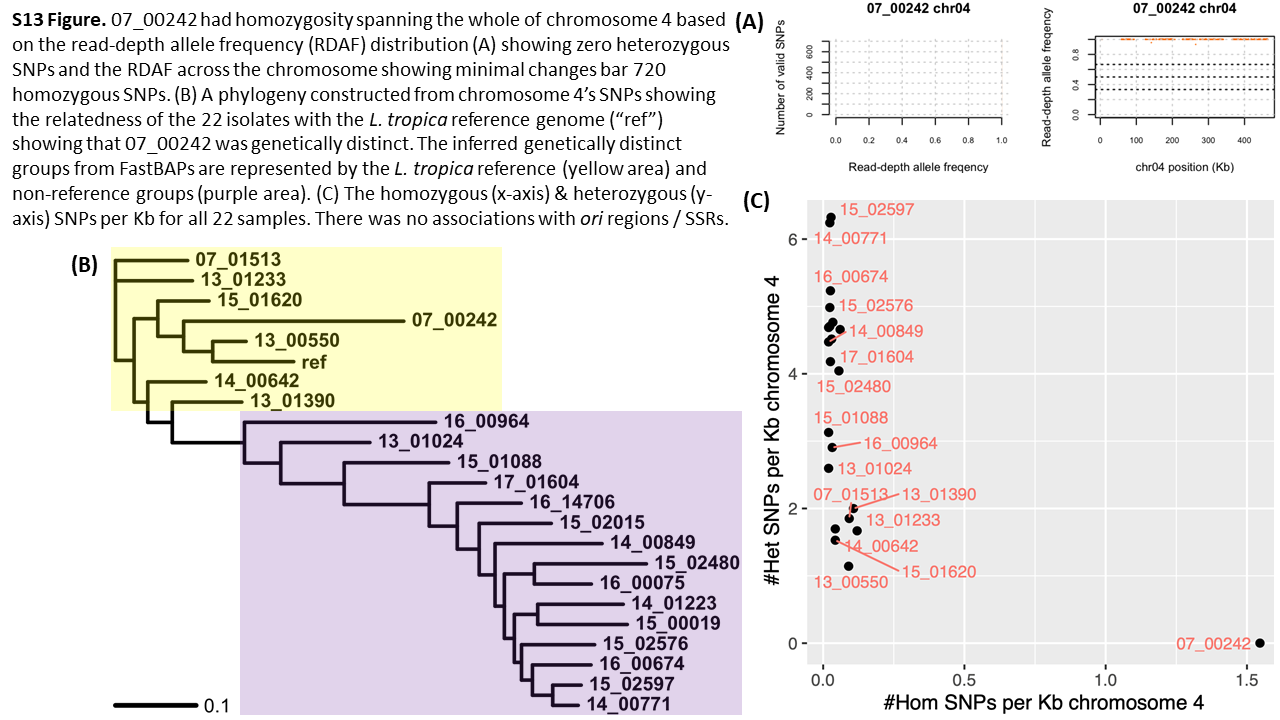

Supplement: S13 Fig — 07_00242 had homozygosity spanning the whole of chromosome 4 based on the read-depth allele frequency (RDAF) distribution (A) showing zero heterozygous SNPs and the RDAF across the chromosome showing minimal changes bar 720 homozygous SNPs. (TIF) [file pntd.0010110.s014.tif]

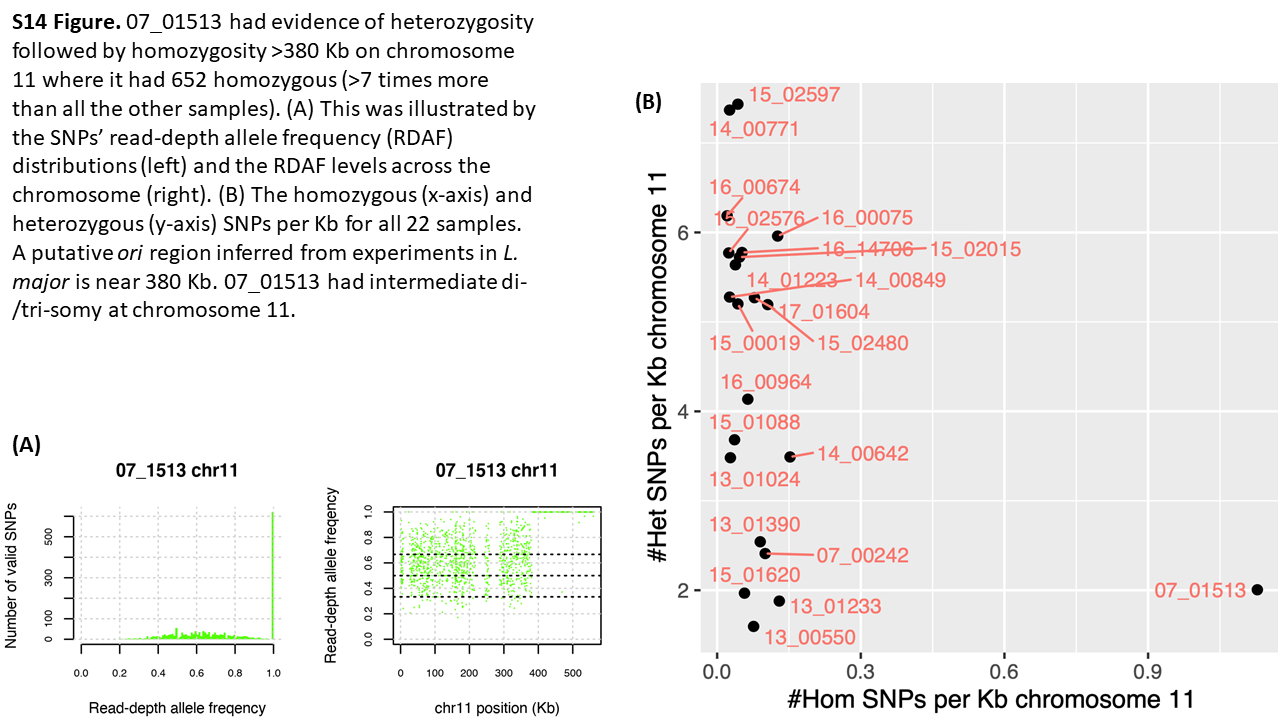

Supplement: S14 Fig — (TIF) [file pntd.0010110.s015.tif]

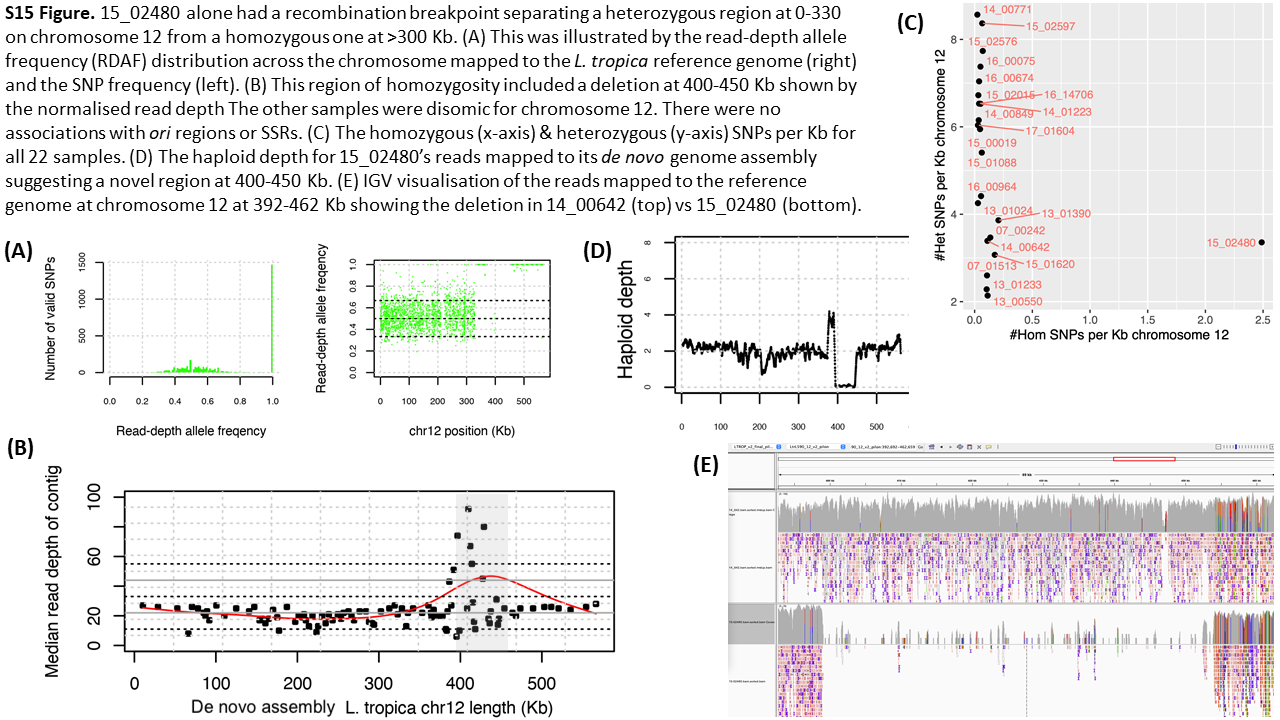

Supplement: S15 Fig — (TIF) [file pntd.0010110.s016.tif]

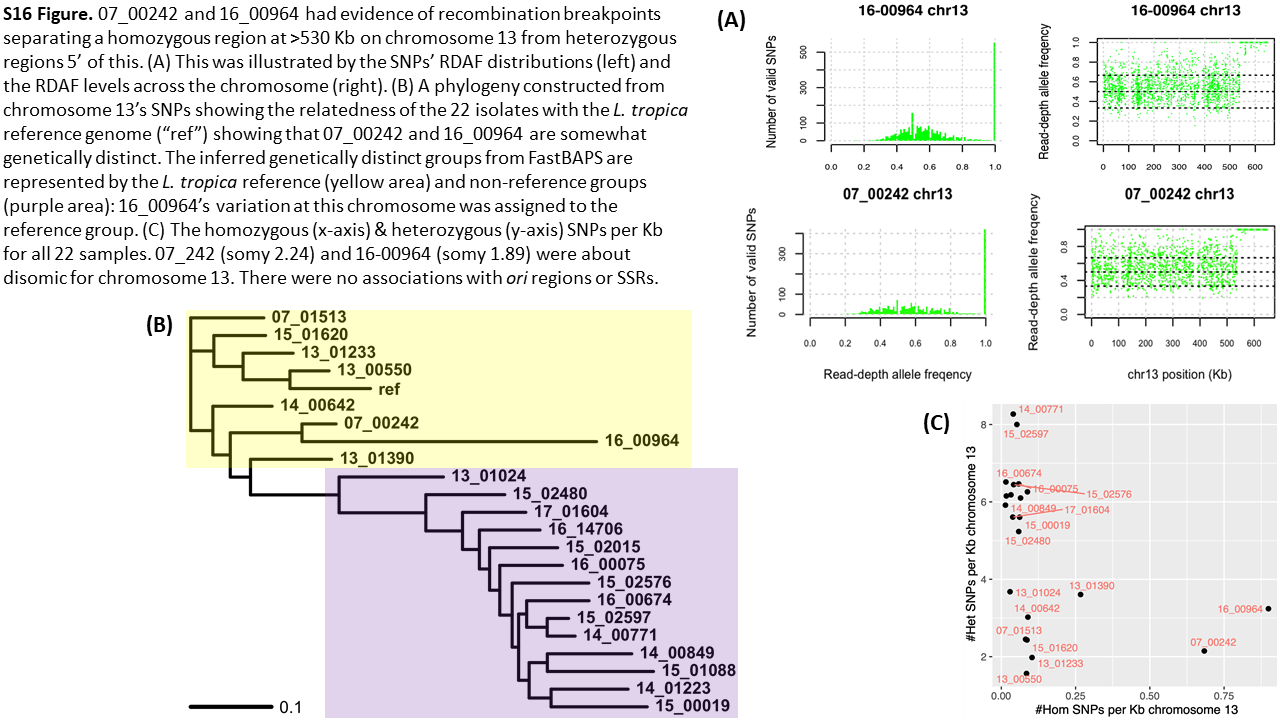

Supplement: S16 Fig — (TIF) [file pntd.0010110.s017.tif]

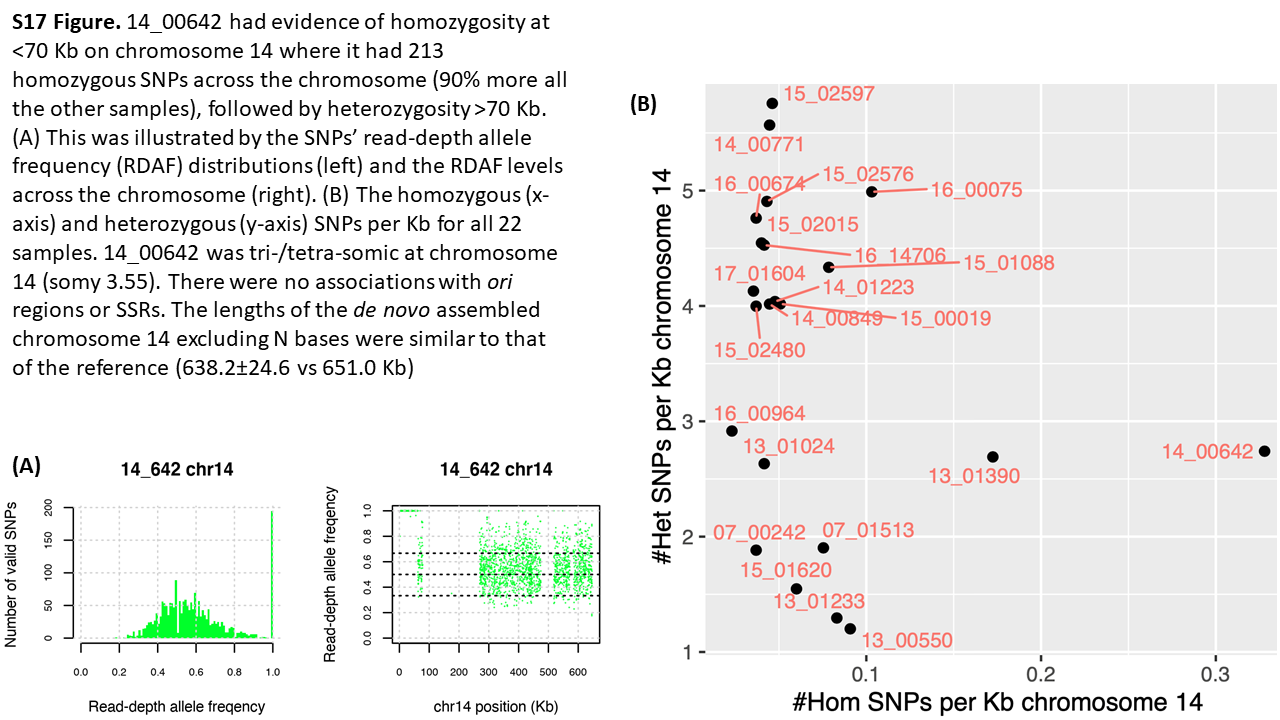

Supplement: S17 Fig — (TIF) [file pntd.0010110.s018.tif]

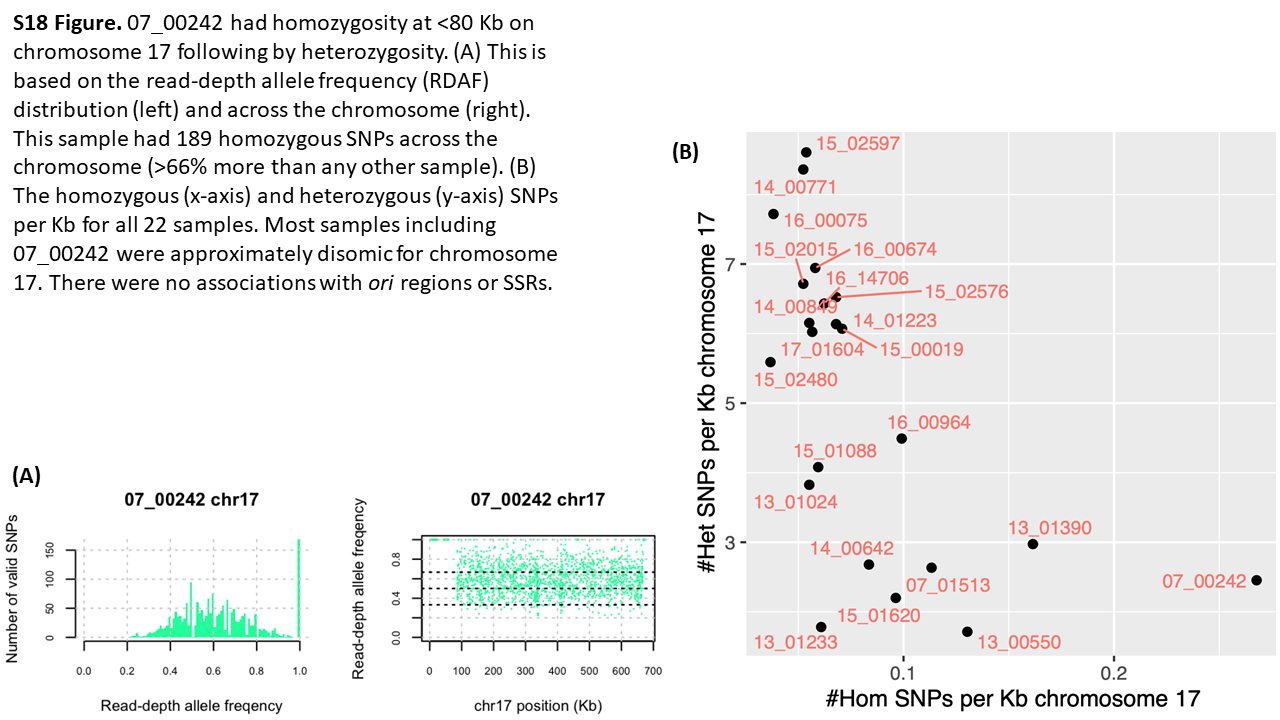

Supplement: S18 Fig — (TIF) [file pntd.0010110.s019.tif]

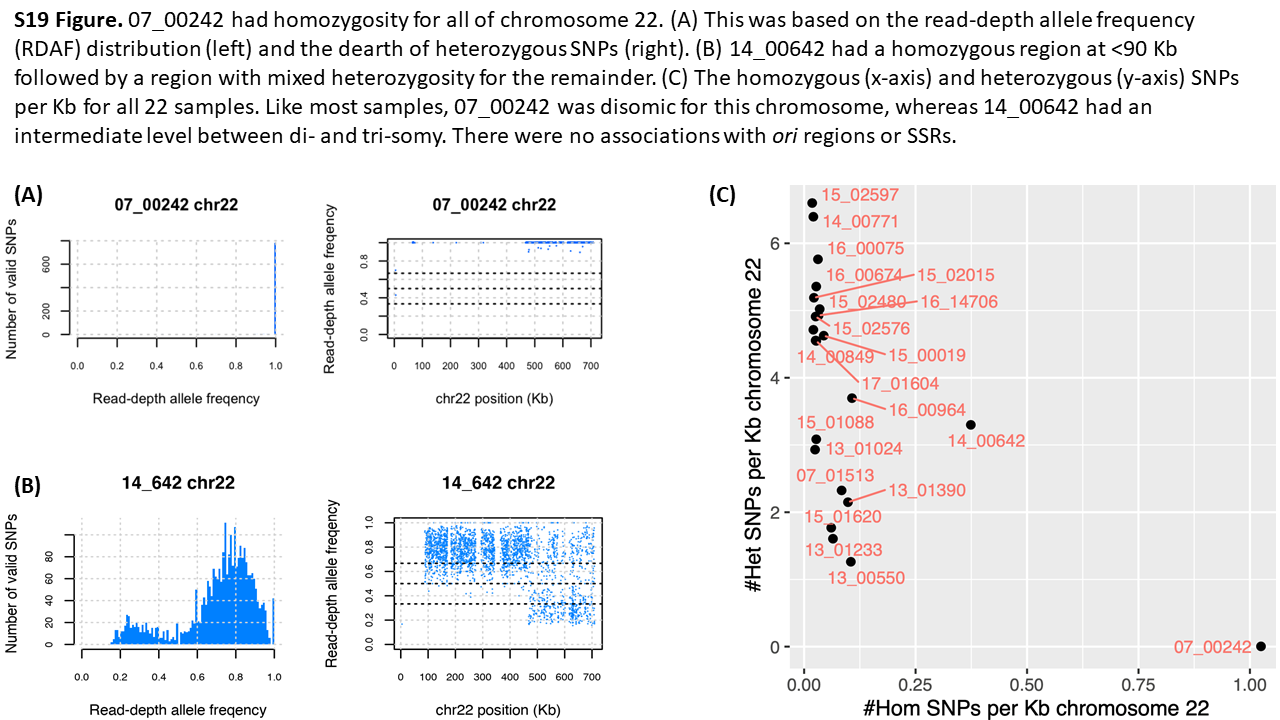

Supplement: S19 Fig — (TIF) [file pntd.0010110.s020.tif]

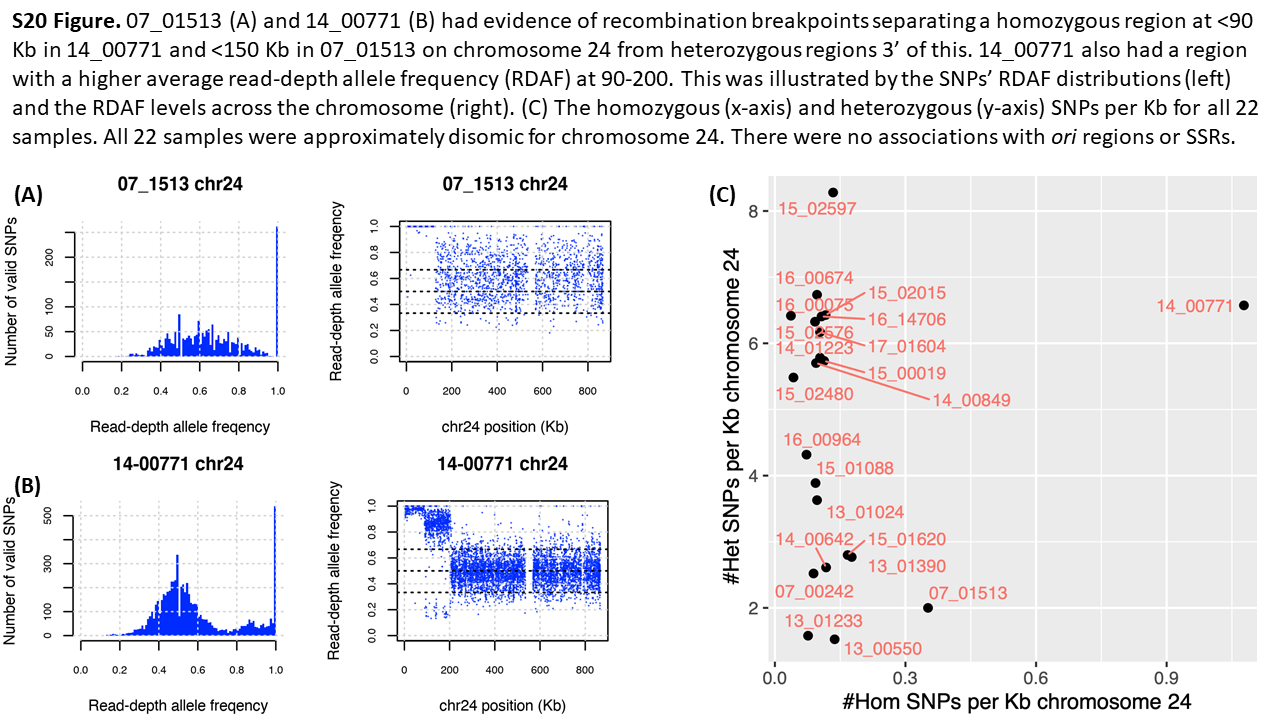

Supplement: S20 Fig — 07_01513 (A) and 14_00771 (B) had evidence of recombination breakpoints separating a homozygous region at <90 Kb in 14_00771 and <150 Kb in 07_01513 on chromosome 24 from heterozygous regions 3’ of this. (TIF) [file pntd.0010110.s021.tif]

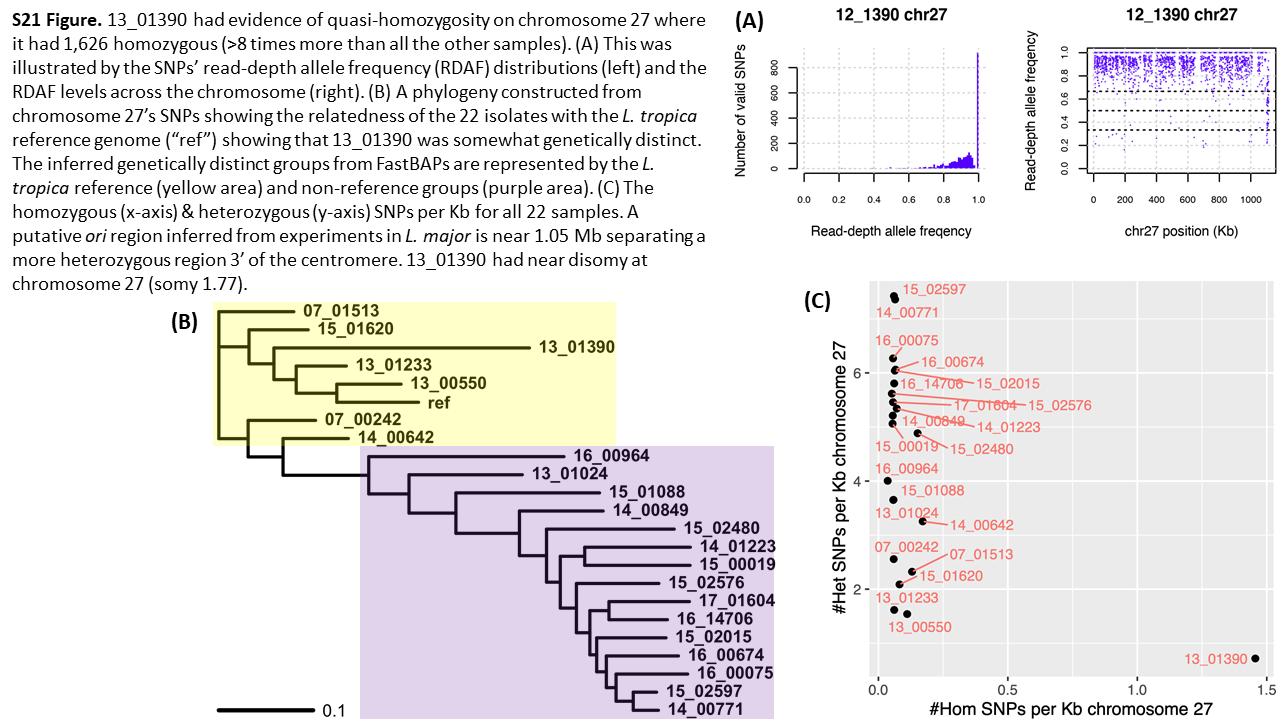

Supplement: S21 Fig — (TIF) [file pntd.0010110.s022.tif]

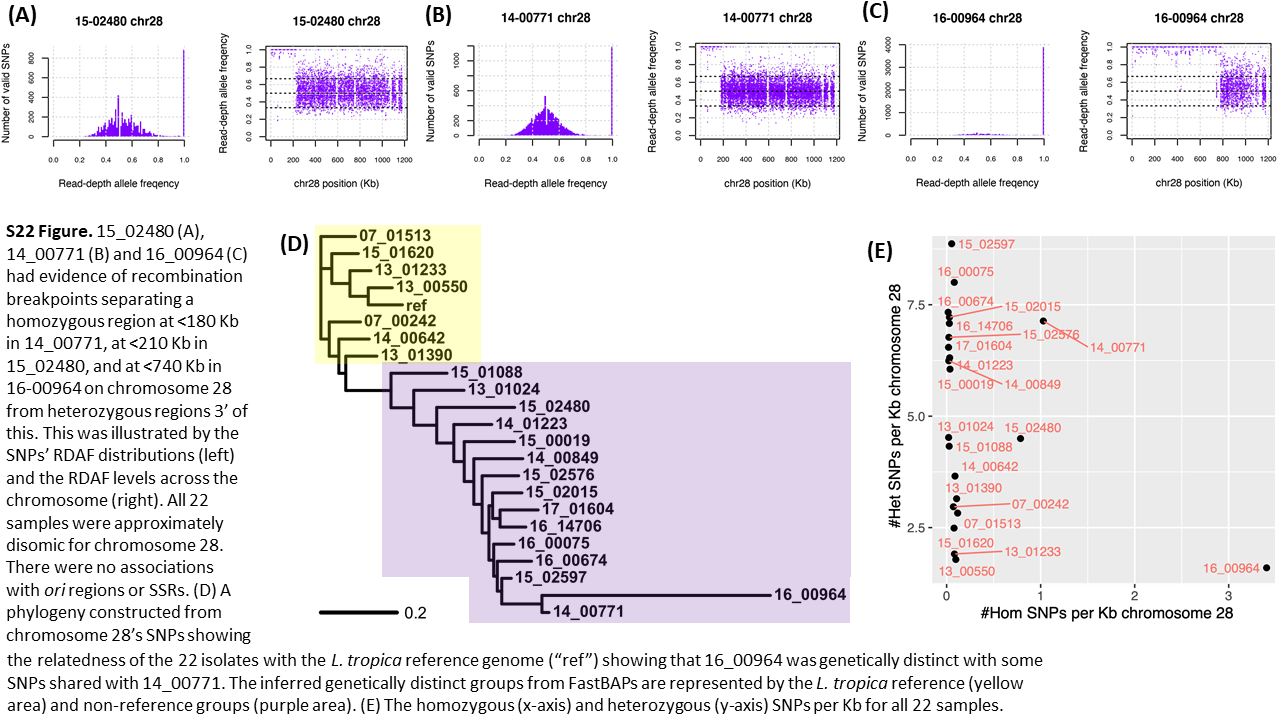

Supplement: S22 Fig — 15_02480 (A), 14_00771 (B) and 16_00964 (C) had evidence of recombination breakpoints separating a homozygous region at <180 Kb in 14_00771, at <210 Kb in 15_02480, and at <740 Kb in 16–00964 on chromosome 28 from heterozygous regions 3’ of this. (TIF) [file pntd.0010110.s023.tif]

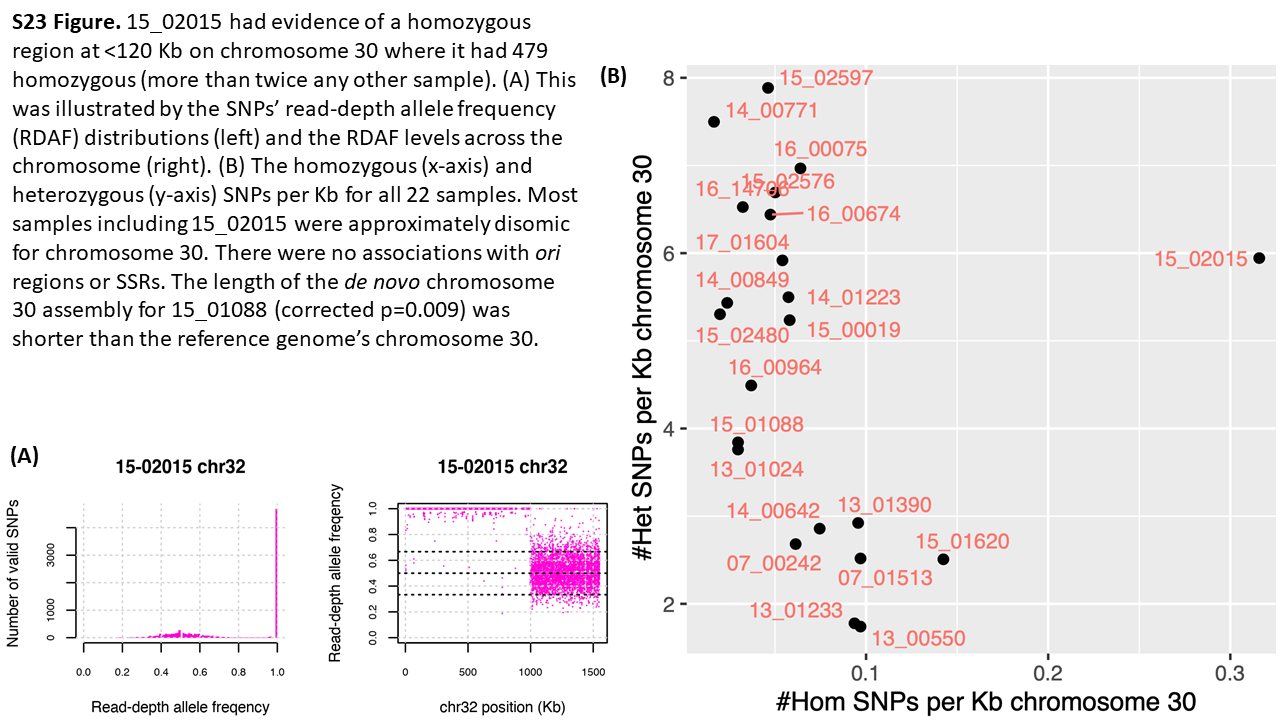

Supplement: S23 Fig — (TIF) [file pntd.0010110.s024.tif]

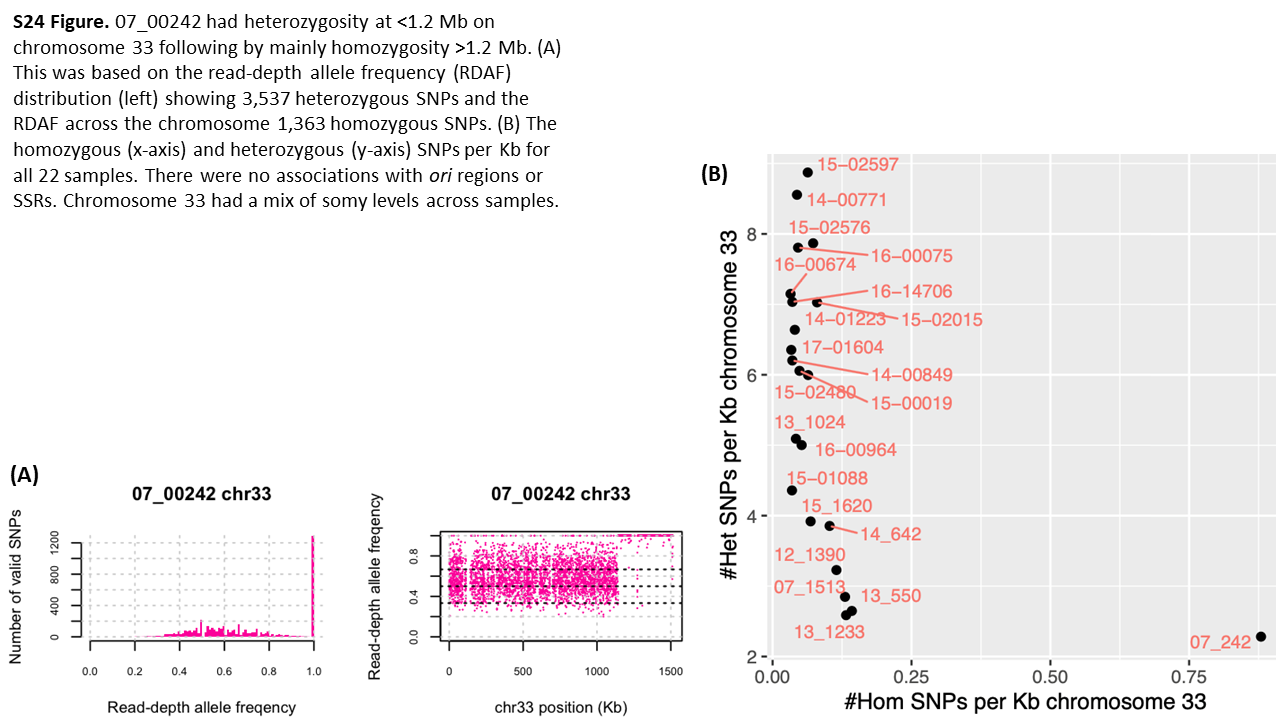

Supplement: S24 Fig — (TIF) [file pntd.0010110.s025.tif]

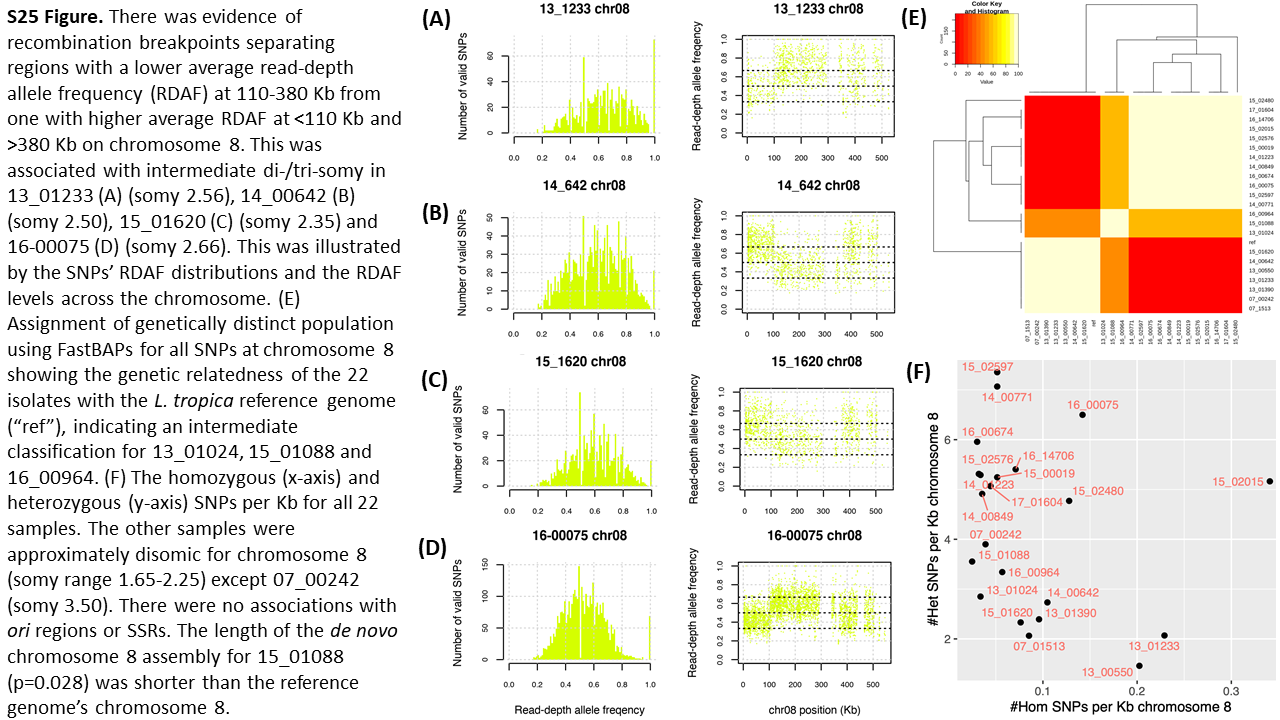

Supplement: S25 Fig — (TIF) [file pntd.0010110.s026.tif]

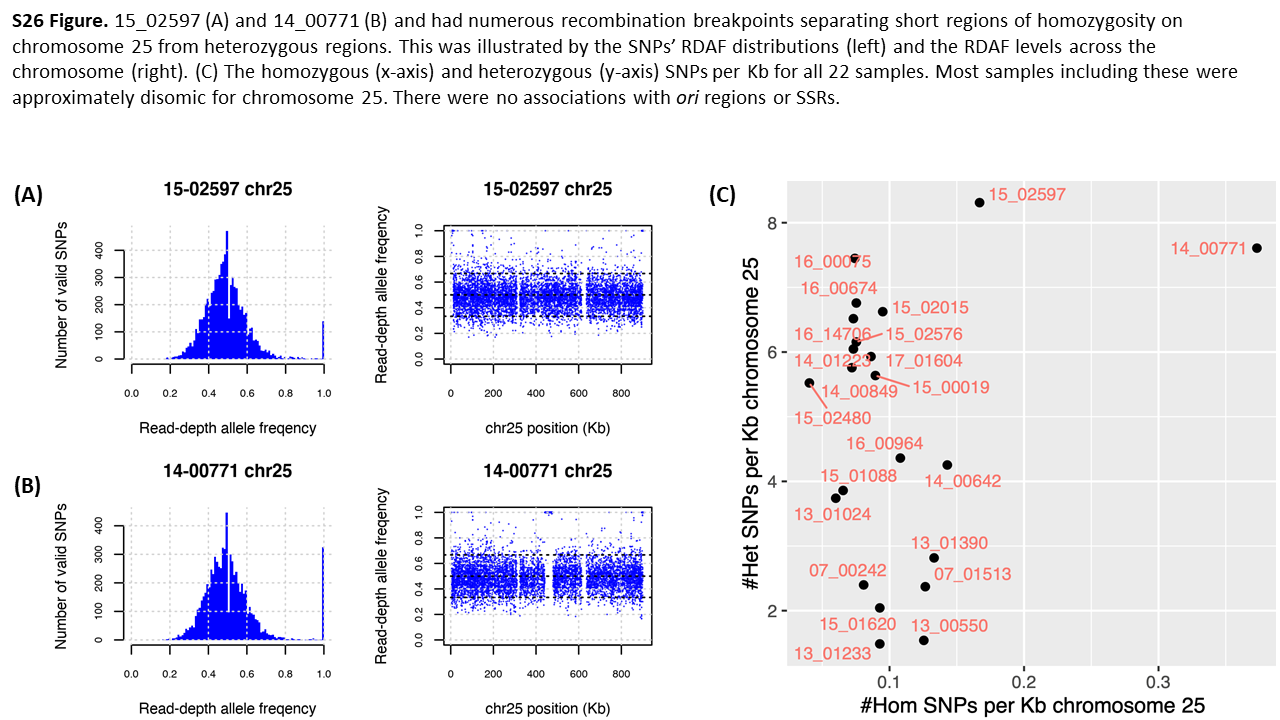

Supplement: S26 Fig — 15_02597 (A) and 14_00771 (B) and had numerous recombination breakpoints separating short regions of homozygosity on chromosome 25 from heterozygous regions. (TIF) [file pntd.0010110.s027.tif]

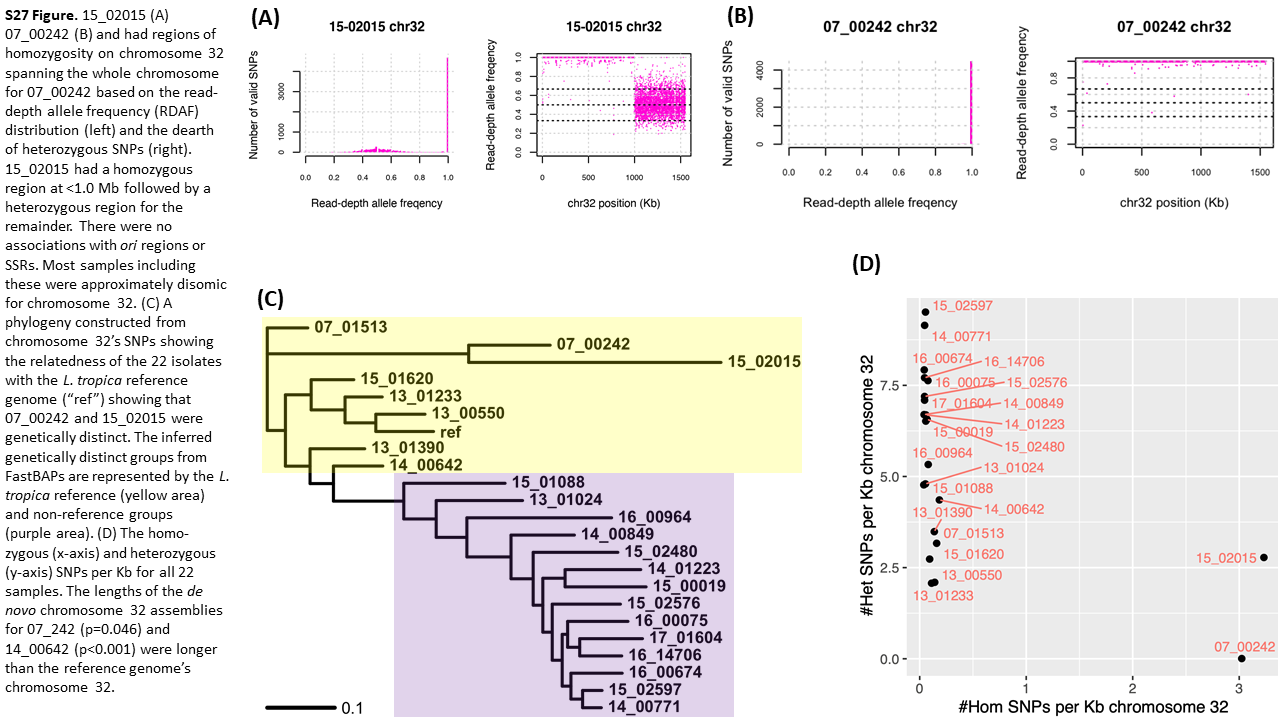

Supplement: S27 Fig — 15_02015 (A) 07_00242 (B) and had regions of homozygosity on chromosome 32 spanning the whole chromosome for 07_00242 based on the read-depth allele frequency (RDAF) distribution (left) and the dearth of heterozygous SNPs (right). (TIF) [file pntd.0010110.s028.tif]

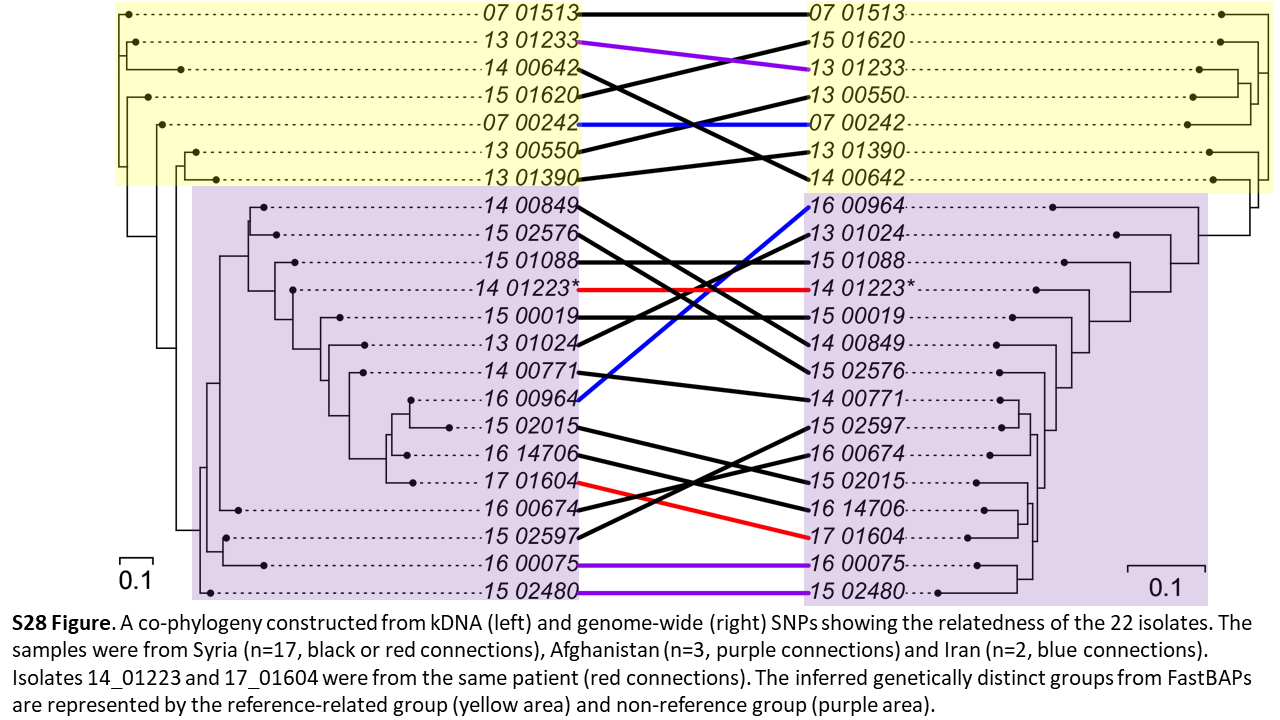

Supplement: S28 Fig — (TIF) [file pntd.0010110.s029.tif]
