# Supplementary material for: High genome plasticity and frequent genetic exchange in Leishmania tropica isolates from Afghanistan, Iran and Syria
Source: PLoS Negl Trop Dis. 2021 Dec 30;15(12):e0010110. doi: 10.1371/journal.pntd.0010110 (PMC8754299; doi:10.1371/journal.pntd.0010110)
Supplement: Attachment — Submitted filename: Point_by_point response PLOS NTD 211109.docx [file pntd.0010110.s030.docx]

Dear Editors*,*

Thank you for your interest in our manuscript entitled “*High genome plasticity and frequent genetic exchange in Leishmania tropica isolates from Afghanistan, Iran and Syria*” (submission ID PNTD-D-21-01154).

We would also like to extend our thanks to the reviewers who provided valuable input and constructive criticism, which we have addressed point-by-point both below and in the revised version of the manuscript. The respective sections that have been modified in the revised version of the manuscript have been marked accordingly.

With these clarifications and corrections, it is our hope that the revised manuscript will be suitable for publication in *PLOS Neglected Tropical Diseases*

Yours sincerely,

Hedvig Glans, on behalf of the authors

**Point-by-point responses to the reviewers’ comments:**

The authors are recommended to carefully address the reviewers' comments and to make available their data to the public as recommended by reviewer 2.

=> We have been in contact with the helpdesk at ENA, the original uploaded files are available through ENA, but the processing has not yet finished.

Meanwhile we have made the sequences available through Zendo through following links; the first 19 FASTQs are available on <https://doi.org/10.5281/zenodo.5645033> and the second 3 FASTQs are available on <https://doi.org/10.5281/zenodo.5647430>

Reviewer's Responses to Questions

<b>Key Review Criteria Required for Acceptance?</b></br></br>
As you describe the new analyses required for acceptance, please consider the following:</br></br>

<b>Methods</b></br></br>
-Are the objectives of the study clearly articulated with a clear testable hypothesis stated?</br>
-Is the study design appropriate to address the stated objectives?</br>
-Is the population clearly described and appropriate for the hypothesis being tested?</br>
-Is the sample size sufficient to ensure adequate power to address the hypothesis being tested?</br>
-Were correct statistical analysis used to support conclusions?</br>
-Are there concerns about ethical or regulatory requirements being met?</br></br>

Reviewer #1: (No Response)

Reviewer #2: (No Response)

Reviewer #3: The authors have used whole genome sequencing for studying population genetics based on chromosomal ploidy and patterns of single nucleotide polymorphisms among a series of 22 L.tropica natural isolates, which is a sound approach.

Reviewer #4: The objectives have not been defined. Lines 142-147 and the entire Methods section refer to the isolation and whole genome sequencing and analysis of 22 Leishmania tropica isolates from patients with the aim of finding genetic diversity and group them in the context of their geographical origin. This design is not appropriate to test the existence of meiosis and parasexual processes in the parasite. No experiment has been planned to test these hypotheses (see comments below).
Ethical approval was received according to the ethics statement in the manuscript, although I don't have access to the document.

= > Ethical approval was obtained from the Central Ethical Review Board in Stockholm (2015/2162–31). Informed consent was not found to be necessary, as the samples included in the genome study were anonymized and only isolated parasites were studied.

Regarding the clinical data, such as treatment and outcome, the Central Ethical Review Board in Stockholm (2015/2162–31) found it not necessary to obtain written consent from the patients to collect retrospective clinical information.

Statistical analysis other than statistical checks in whole genome sequence analysis is not applicable here because only chromosomal sequence patterns are being addressed.

=> We understand the reviewer's perspective and have addressed their more detailed comments.

--------------------

<b>Results</b></br></br>
-Does the analysis presented match the analysis plan?</br>
-Are the results clearly and completely presented?</br>
-Are the figures (Tables, Images) of sufficient quality for clarity?</br></br>

Reviewer #1: (No Response)

Reviewer #2: (No Response)

Reviewer #3: The authors are transparent with their data as shown by the high number of supplementary files which are of sufficient quality. The quality of the original figures is likely not enough for publication (they appear fuzzy).

=> To improve the quality, the figures were submitted through the Preflight Analysis and Conversion Engine (PACE).

Reviewer #4: The results are well presented, match the analysis plan and show some examples of recombination and patchy heterozigosity, aneuploidy and structural variation. However, the experiment is not well planned to address the existence of meoisis and parasexuality in Leishmania (see comments below). 
Some panels in figures are too small to be read (axis titles and scales, for example).

=> The figures and their panels have been altered to improve the quality and to make them easier to read. The comments on meiosis and parasexuality are addressed below.

--------------------

<b>Conclusions</b></br></br>
-Are the conclusions supported by the data presented?</br>
-Are the limitations of analysis clearly described?</br>
-Do the authors discuss how these data can be helpful to advance our understanding of the topic under study?</br>
-Is public health relevance addressed?</br></br>

Reviewer #1: (No Response)

Reviewer #2: (No Response)

Reviewer #3: The conclusion of the paper ressembles more of an introduction than a conclusion. Their data is interesting and support genetic exchange. However it do not explain the adaptation of Ltropica to different vector species nor do it explain the challenges in treating CL by Ltropica. No such link with their data was made.

=> We have rewritten the conclusion as follows;” *The high diversity, frequent changes in heterozygosity and abundant aneuploidy in these 22 L. tropica isolates were consistent with genetic exchange, possibly by sexual or parasexual mechanisms. Different frequencies of recombination have been observed between different Leishmania populations (80). L. tropica - L. donovani hybrids that have spread to geographically distant regions though diverse Phlebotomomus spp. by genetic exchanges to adapt to the vector have been described (16, 28, 89). It remains unclear if this is due to intrinsic differences in Leishmania or due to differences in vector species. What is clear is that L. tropica has adapted to several vector species, which seems to increase hybridization, and can produce new progeny with genetically highly similar or extremely distinct Leishmania. The diverse ancestries, mixing and existence of hybrids may help explain the challenges in treating CL and leishmaniaisis revidans caused by L. tropica in the Middle East, but further research needs to be carried out to better understand the correlations between genetic diversity in Leishmania, the differences among the vectors and treatment outcome* “.

Reviewer #4: The conclusions are speculative. The patchy heterozygosity patterns found just suggest but not definitely support the existence of meiotic and parasexual processes. The results point to important recombination processes, but other recombination mechanisms based on mobile genetic elements that may explain such events have been described. For example, those based on the Pr77 hallmark. Also, aneuploidy has been associated to chromosome amplification as a mechanism of gene expression modulation. For example, it has been described in antimony-resistant strains. In fact, all patients included in the study to obtain the isolates were treated with different outcomes (cure, relapse or failure). Each clinical situation and patient-parasite interaction could have resulted in different amplification patterns or recombination processes under the selective pressure. The reduced number of samples makes addressisng these issues difficult. Therefore, the suggestions presented as
conclusions have not been experimentally proven. At this preliminary stage, the results do not reach sufficient public health relevance. –

=> As the reviewer suggests, additional studies would be required to definitely prove the occurrence of meiotic and parasexual processes. In accordance with this, we have rewritten key sentences and toned down the conclusions.

Mobile elements are much less abundant in *Leishmania* compared to *T. cruzi* (doi: 10.1186/s12864-016-2427-6)*,* where the suggested recombination mechanism was studied. This makes it more unlikely that this mechanism is responsible for the patterns that we have observed in *L. tropica*. We have nevertheless added a sentence to the manuscript that mentions this possibility: “*Recombination involving mobile genetic elements have also been suggested as a possible mechanism to the mosaic aneuploidy*”.

--------------------

<b>Editorial and Data Presentation Modifications?</b></br><br/>
Use  this section for editorial suggestions as well as relatively minor modifications of existing data that would enhance clarity. If the only modifications needed are minor and/or editorial, you may wish to recommend “Minor Revision” or “Accept”.

Reviewer #1: (No Response)

Reviewer #2: (No Response)

Reviewer #3: 1- There is a problem with Figure S4: the 3rd and 4th rows are the same (3rd row is repeated in the 4th row) and some strains are missing from the figure; this should be corrected.

=> We thank the Reviewer for spotting this, it has been amended in the revised version.

2- Line 29: 'where IT is known...'

=> Thanks for your comment. The word “it” is added to the sentence

3- Line 33: 'tropica' should be in italic

=> Thanks for your comment. The word “tropica” is now in italic.

4- Lines 57 and 61: 'species' should not be in italic

=> Thanks for your comment. The word “species” on line 57 and 61 are now not in italic.

5- Line 73: change to 'Phlembotmine' to 'Phlebotomine'

=> Thanks for your comment. The word is now changed to “Phlebotomine”.

6- Lines 74 and 76: change 'serengeti' to 'sergenti'

=> Thanks for your comment. The word “serengeti” is changed to “sergenti”.

7- Line 75: The beginning of the sentence up to 'zoonically' is not clear, should be rephrased.

=> Thanks for your comment. We have rephrased the sentences to make it more clear, as follows “*In most geographic areas, L. tropica is transmitted by its most common vector, the female Phlebotomus sergenti sandfly (17). Although other vectors like P. arabicus in northern Israel (18) and P. guggisbergi in Kenya (19) also are known*”.

8- Line 154: 'fluconazole'

=> Thanks for your comment. “Fluconazole” is now correctly spelled at line 154.

9- Line 157: 'second sample IN 2017'

=> Thanks for your comment. The word “in” is now added to the sentence.

10- Line 179: 'at 23oC ' instead of in 23oC

=> Thanks for your comment. The word “in” is now exchanged to “at”.

11- Line 184: please define 'MGB'

=> Thanks for your comment. TaqMan MGB is a commercial nomenclature. MGB stands for minor groove binder. We have added this information as follows “*The libraries were quantified by an in-house qPCR, MGB (minor groove binder) assay (64)*”.

12: Line177: no need to mention the samples that were discarded as these are not further discussed nor is an explanation provided for the low quality of their libraries.

=> On line 177, there are no comments on the low quality of the library. Line 197, on the other hand, mention “*differences in library-specific qualities that eliminated samples*” which have been removed, as follows “*The reads were trimmed including removal of adaptor sequence using the Torrent Suite software with standard settings (Torrent suite v5.2.2) (SRA accession PRJEB45563) resulting in 22 valid short read libraries*”.

13- Line 197: There is a lot of variability in the percentage of discarded reads between libraries and the authors should briefly discuss about the possible reasons explaining this difference in sequencing quality.

=> To address this, we added a sentence “*The quality of the initial DNA read libraries varied considerably, so careful quality control was conducted to ensure only high-quality reads were retained and low-quality reads were discarded (Table S1)*” after the 1st sentence of the paragraph for the Methods section “Read processing, mapping and SNP screening”.

14- Line 210: from FigS1B their is not benefit of using MQ30 on the number of called SNPs. MQ55 or MQ60 looks more appropriate. Please described why MQ30 was used and how it was selected as a cutoff value.

=> Although the base quality scores were quite variable, the mapping quality scores were uniformly high as illustrated in Fig S1B. It is unlikely that those reads were mapped incorrectly with a MQ > 50 (ie, P(error) = 0.00001). MQ > 30 is conventional (eg 10.1371/journal.pcbi.1008678) across diverse papers, and additionally a higher level would not have made a major difference to the results obtained.

15- Line 269-270: a parenthesis is opened at line 269 but is not closed (should be closed after ref 76)- => Thanks for your comment. There is now a “)” after the reference.

16- Lines 274-276: It is not clear what these SNPs are. It is mentioned that 1594 SNPs were detected among the 22 samples but the next sentence says that '220 SNPs were found within our collection'. This seems inconsistent, please clarify.

=> We re-phrased those sentences to be more clear: “T*his found 1,594 SNPs distinguishing* ***all of*** *the 22 isolates from the L. tarentolae reference, which phylogenetically clustered most closely with isolate 13_01390. Within the 22 L.tropica alone, there were 220 variable sites, with a frequency per sample ranging from 9 to 154 SNPs with an average of 82±39 SNPs.*”

17- Line 284: at line 190 the author mentions >30-fold coverage. Please be consistent and correct for the proper coverage value.

=> We modified this to “*>30x genome-wide coverage*”.

18- Line 288: define 'SVs'

=> Thanks for your comment. “SVs” stands for structural variants and to define this the sentence has changed as follows: “Th*is yielded power to examine large structural changes of >10 Kb: small structural variants and indels were not examined in detail here*.”

19- What do the authors mean by 'artefacts associated with the sequence quality'; on which criteria?

=> This sentence was phrased poorly and has been amended as above.

20- Line 308: for clarity please please specify the reference genome. I know it is mentioned in the Methods but this will help the reader appreciate what was done if repeated here (especially that later in the same paragraph the authors mention an alignment on a distinct reference from the assembly of 13_00550).

=> We clarified that we are using the “*L. tropica LRC-L590 reference genome*”.

21- Line 315: 'sequencing OF isolate'

=> Thanks for your comment. The word “*of*” is added to the sentence.

22- Line 316- 'like the other 21 isolates'. It is not clear to the reviewer whether the 21 isolates were also mapped to the 13_00550 assembly or if the authors are referring to the number of heterozygous SNPs detected from the alignment of the 21 isolates against the L590 genome.

=> We clarified this to say “... *high heterozygosity compared to when the reads were mapped to the L. tropica LRC-L590 reference genome*”.

23- Line 370: 'that may possibly have affected the treatment process'. This is highly speculative. Are there SNPs that would support such claim? In genes reputed to be implicated in treatment failure? or whose function could possibly be linked with drug response?

=> Thanks for this helpful comment. We agree and have removed this last part of the sentence, as follows “*The patient had a mixed infection with two unique isolates, allocated to the non-reference genetic group, but with clear genomic differences*”.

24- Lines 387-388: this is data interpretation and should not be part of the figure caption, which is about describing the figure and not discussing on its data. – Thanks for your comment. We have rewritten the figure caption regarding 3A as follows: “*(A) Patterns of heterozygosity (blue), a LROH (grey) and homozygous similarity (orange) are seen in 14_01223, 07_00242 and 16_00964 (green), and high heterozygosity in 16_00075 and 14_642 (red)*”.

25- Line 426: From FigS7 chr 23 seems to be the one with the lower RDAF,  not chr 31.

=> Yes we had noted that in the legend where the plot focused on heterozygous SNPs (“Chromosome 23 had a lower RDAF rate (0.43±0.06) compared to the other chromosomes (average 0.52±0.04)”) whereas the text had focused on all SNPs: “*the RDAF distribution of chromosome 31 across all SNPs (0.50±0.05 per sample) was the lowest of all chromosomes (average 0.56±0.06)*”. For clarity, we amend this to say “*The RDAF distribution of chromosome 31 of heterozygous SNPs was second lowest of the chromosomes (Fig S7)*”.

26- Line 494: Shouln't it be Fig 6 instead of Fig 5?

=> Thanks for this helpful comment. It should be Fig 6 and not Fig 5. We have changed it.

27- Line 496: From FigS7, chr23 has the lowest RDAF not the highest.

=> Yes that Fig S7 showed the heterozygous SNPs RDAFs, rather than that of all SNPs as per that text: “... *the highest mean RDAF across all SNPs (0.63±0.06 per sample) of all the chromosomes (average of the other 35, 0.56±0.06)*”. Consequently, we have amended that text to say “.. *the lowest mean RDAF using heterozygous SNPs (0.43±0.06 per sample) of all the chromosomes (average of the other 35, 0.52±0.04)*”.

28- Line 527: 13_0050 instead of 13_550

=> Thanks for your comment. The isolate identification number is now corrected to 13_00550.

29- Lines 553-558: 'such as both derived alleles were present' what does this mean exactly? The same for '...the most interesting LROHs...were parralleled by more rare...' what do they authors mean by parralleled?

=> “*Both derived alleles*” was clarified as “*two derived alleles*”. The 2nd component was modified to say “*In addition to these unusual LROHs at chromosomes 10, 23, 29, and 36 above, we observed rare switches …* ”.

30- Line 593: What does 'All bar' means?

=> It means “*all except*”. We have amended this to the latter in the submission.

31- Line 599: Here is a good place to discuss about the sequencing quality I mentionned earlier. What does the data miss to be able to address these questions?

=> Although our reads were mainly 200-400 bases long, they were not paired-end, and so their resolution may have been limiting. Consequently we amended that sentence to start with: “*Due to the effects of PCR during library preparation and the single-ended nature of these read libraries, they were insufficient to …*”. The read coverage is too variable to be confident about exact breakpoints in coverage, and paired-end / mate pair / Nanopore libraries might be better able to resolve these differences.

Reviewer #4: Clarity in some figure panels should be improved.

=> To improve the quality the figures were submitted through the Preflight Analysis and Conversion Engine (PACE). The level of resolution should now be improved and the online supplementary material allows for more detailed viewing by the readers.

--------------------

<b>Summary and General Comments</b></br></br>
Use  this section to provide overall comments, discuss strengths/weaknesses of the study, novelty, significance, general execution and scholarship. You may also include additional comments for the author, including concerns about dual publication, research ethics, or publication ethics. If requesting major revision, please articulate the new experiments that are needed.

Reviewer #1: This well written paper provides a sound whole genome sequencing analysis of 22 strains of Leishmania tropica.   The main conclusions drawn regarding genomic diversity of the strains that may reflect genetic exchange by sexual or parasexual processes are supported by the data and are consistent with prior studies on L. tropica and other Old World strains.   While the findings may not represent a major advance,  the report will nonetheless provide the field with  informative new datasets that have been carefully analyzed and displayed by the application of  currently available bioinformatic tools.  
There are a few minor points that can be addressed:

Lines 80-81:   …..subsequently as amastigotes within mammalian  monocytes (23).
NOT correct

=> Thanks for your comment. We agree and have altered monocytes to macrophages. The new sentence as follows, ”.*.. and subsequently as amastigotes within mammalian macrophages* (23)”

Lines 84-85:  Evidence of meiosis and hybridisation has been observed in diverse natural isolates of Leishmania (25-  28 )
These studies provide evidence for hybridization, not meiosis.   The mode of genetic exchange could not be inferred from these studies. -s.

=> Thanks for this helpful comment. We agree and have removed the comment on meiosis as follows, “*Evidence of hybridisation has been observed in diverse natural isolates of Leishmania* (25-28 )”.

Lines 85-86:  Reproduction in Leishmania is facilitated by its genes for meiosis prometaphase 1 (24) to allow crossing-over, resulting in recombinant chromosomes (30).  
There is as yet no evidence that any of the meiotic gene homologues in Leishmania are functional or play a role in genetic exchange.

=> We have adjusted the sentence to acknowledge this uncertainty: “*It is possible but still uncertain that reproduction in Leishmania could be facilitated by genes with high similarity to ones involved in meiosis (24) to allow crossing-over, resulting in recombinant chromosomes (30)*”

Legend to fig  2b and c are incorrect, need to be interchanged.

=> This was amended (“*(B) 14_01223 had a region with a high RDAF at 1.63-1.78 Mb followed by homozygosity at >1.80 Mb that 17_01604 did not have. (C) Chromosome 20 was approximately trisomic in 14_01223 but disomic in 17_01604 as shown by the RDAF distributions.*”).

The description of the two genetically distinct strains isolated from the same patient is very interesting.  Since the genomes remain highly homologous, Is it possible that instead of a mixed infection, the genomic differences observed reflect selfing between the same strain that occurred in the vector, with simultaneous transmission of selfing and non-selfing promastigotes?   Since genetic exchange involving intracellular amastigotes has recently been described (Telittchenko and Descoteaux, 2020), it is also possible that a selfing event occurred in ther
human host.

=> While we acknowledge this paper on promastigotes and amastigotes in *L. mexicana* and *L. amazonensis* and possible intraclonal genetic exchange is very interesting and links to our work. We would expect for a mixed infection a deviation in the read depth allele frequencies (RDAFs) per chromosome away from the expected values based on the somy copy number (diomic, trisomic, etc). However, we did not observe the latter. We did observe extensive genetic differences between the two isolates comparable to differentiation among the other isolates from other patients, indicating a lack of direct evidence of hybridisation among these two specimens.

Line 632:  Effect of long-term culturing of Leishmania and the environmental condition in vitro have previously  been studied (ref).
Reference needs to be included

=> Thanks for this important comment. The references have been included.

Reviewer #2: General comments
The authors report here a genome-wide SNP analysis of 22 isolates of Leishmania tropica, revealing extensive genomic variation and a very complex population structure with evidence of genetic exchange. While the results are consistent with previous observations, clear examples of different ancestries on same chromosomes is shown through a careful dissection of changes in heterozygosity.
While the study is limited in scope, not allowing the authors to examine genetic variation and phylogenies in the context of important metadata such as treatment outcome, it is carried out soundly and with robust methodology and data analysis.

Minor comments/corrections

Ln 111 – The wording suggests that Leishmania regulates gene expression through gene dosage (chromosome and gene copy variation) This may be a little misleading considering the significant amount of post-transcriptional regulation

=> Thanks for your comment. The sentence is rewritten as follows, “*Together with post-transcriptional regulations, Leishmania exploits gene dosage by chromosome and gene copy number variation* (46-48) *which are driven by variable environments that maintain expression levels and genetic diversity* (49). “

Ln 182 – replace ‘were’ with ‘was’

=> Thanks for your comment. The word “*were*” is now replaced with “*was*”

Ln 186 – Bioproject PRJEB45563 is not public and there are no sequences associated with it. Data should be released before publication.

=> We have been in contact with the helpdesk at ENA, the original uploaded files are available through ENA, but the processing has not yet finished. They are working on it.

Meanwhile we have made the sequences available through Zendo through following links; the first 19 FASTQs are available on <https://doi.org/10.5281/zenodo.5645033> and the second 3 FASTQs are available on <https://doi.org/10.5281/zenodo.5647430>

Fig 2. Labels for panels B and C are switched

=> This was amended (“*(B) 14_01223 had a region with a high RDAF at 1.63-1.78 Mb followed by homozygosity at >1.80 Mb that 17_01604 did not have. (C) Chromosome 20 was approximately trisomic in 14_01223 but disomic in 17_01604 as shown by the RDAF distributions.*”)

Ln 370 – ‘possible’ should be ‘possibly’

=> The sentence is re-written.

Reviewer #3: 1- Line 197: There is a lot of variability in the percentage of discarded reads between libraries and the authors should briefly discuss about the possible reasons explaning this difference in sequencing quality.

=> To address this, we added a sentence “*The quality of the initial DNA read libraries varied considerably, and careful quality control was conducted to ensure only high-quality reads were retained and low-quality reads were discarded (Table S1)*” after the 1st sentence of the paragraph for the Methods section “Read processing, mapping and SNP screening”.

2- Line 210: from FigS1B their is not benefit of using MQ30 on the number of called SNPs. MQ55 or MQ60 looks more appropriate. Please described why MQ30 was used and how it was selected as a cutoff value.

=> Although the base quality scores were quite variable, the mapping quality scores were uniformly high as illustrated in Fig S1B. It is unlikely that those reads were mapped incorrectly with a MQ > 50 (ie, P(error) = 0.00001). MQ > 30 is conventional (eg 10.1371/journal.pcbi.1008678) across diverse papers, and additionally a higher level would not have made a major difference to the results obtained.

3- Lines 213 and 306: 301659 SNPs is lower than the sum of the Candidate_SNPs column in TableS1. I guess this is because SNPs shared by different samples are counted as one SNP. If so please be mention this explicitely (either at line 213 or 306) and it would be nice to have a global idea of the level of SNPs redundancy between samples.

=> We clarified to say that this was “*301,659 valid SNPs across all samples*” and “*301,659 SNPs across all samples*” respectively.

4- Line 222: What are these outlying chromosomes with extreme SNPs rates?

=> We applied t-tests and other statistical approaches that assume the data is Normally distributed. In certain instances when comparing a single chromosome with an extreme SNP density, it was compared to the other 35, and hence that single chromosome was outlier with an extreme SNP rate.

5- Line 313: Samples 16_00964 and 07_00242 seems to bring a lot of weight in the negative correlation. If removing these outliers there seems to be 2 groups having roughly the same number of homozygous SNPs but differenciated by the number of heterozygous SNPs. Is the correlation still holds when excluding the 2 outliers?

=> The reviewer is correct that there is no real correlation present. The intention of illustrating the small negative correlation is to highlight how this contrasts with the expectation from genetic drift of a positive association between the numbers of heterozygous and homozygous SNPs per sample. Hence in the paper we continue to refer to this as “.*.. suggesting processes other than genetic drift were contributing to the variation*”.

6- Lines 325-328: Why is sample 16_00964 labelled in Figure 1 as part of the non-ref group if about half of its chromosomes ambiguous? What is the statistics supporting its assignement to this group?

=> FastBAPS allocated that samples to the non-reference group based on the overall genome-wide SNP rates, and (as mentioned in the text) on a per chromosome basis 24 out of 36 chromosomes were consistent with that assignment (“*12 out of the 36 chromosomes of 16_00964 were allocated to the reference group by FastBAPS and 24 to the non-reference genome group*”). In the context of this paper, understanding when and why chromosomes in any sample had inconsistent group assignments was of key interest as examined later.

7- Line 344: the authors should explicitly mention the number of shared SNPs between samples 14_01223 and 17_01604. For now only the total number of SNPs in each of these samples is indicated. Also how much of the non-shared SNPs part of chromosomes 2 and 36? This would help the reader appreciate how much the heterozygosity of 17_01604 for these 2 chromosomes accounts for in the difference between these 2 samples isolated from the same patient.

=> The number of homozygous SNPs between 14_01223 and 17_01604 was 11,885, and this was 65,616 for heterozygous SNPs. At chromosome 2 alone, that was 254 homozygous SNPs and 1,616 heterozygous SNPs. At chromosome 36, that was 5,580 homozygous SNPs and 15,606 heterozygous SNPs. We have revised this in the paper, where we now say: “*These two isolates were genetically different from each other with no evidence of a mixed infection (Fig 1): 14_01223 and 17_01604 had 159,535 genome-wide SNPs between them, including 1,616 on chromosome 2, and 15,606 on chromosome 36”.*

8- The results section is sometimes confusing because some data is repeatedly described. For example the heterozygosity of chr36 in 14_01223 mentionned at lines 365-366 had already been mentionned at line 361-362 and it at first confusing whether this is a new piece of information of not. Another example is the sentence at lines 411-413 which is a repeat on the zygocity pattern of 16_00075 which had been described in the sentence just before (lines 408-410). Another example is the paragraph from lines 445 to 454. the authors start by describing RDAF profles for chr 31 in 4 samples, mentionningthe positions affected and the ID of the samples. then they come back after with more details for each samples by mentionning again the same positions. This is a lot of nucleotide IDs and RDAF for the reader to process. The authors should try to improve how they are conveying the data and associated interpretation throughout the Results section. For example for the later, it would be simpler to start the paragraph by saying that changes in heterozygocity were observed for samples A, B, C and D. then continue with the specific RDAF values and chr positions for each samples, and finish by indicating which of these positions are associated with TSS or other genomic features.

=> To address the reviewer’s input, we removed “*at a strand switch inferred from L. major*” from the Figure 2 legend (on 14_01223). And we removed “*16_00075 had a breakpoint at an acH3/BaseJ site at 1.28 Mb*” from the Figure 3 legend. We removed “*Chromosome 31 was tetrasomic in all except 15_02597, which was pentasomic (middle)*” from the Figure 4 legend. As suggested, we modified that paragraph on chromosome 31 to read as: “*Chromosome 31 had sharp changes in heterozygosity at 220 Kb and 950 Kb in samples 15_02015 and 15_01620, and at 950 Kb in 15_02597 (for which chromosome 31 was pentasomic) (Fig 4). In 15_01620, the major RDAF switched from a mode of 0.75 at <220 Kb to 0.5 at 220-950 Kb, before increasing to 0.75 at >950 Kb (Fig 4). In 15-02576, the major RDAF modes were 0.4 and 0.6 <950 Kb, followed by 0.6 at >950 Kb (Fig 4). 15_02597’s RDAF had a major peak of 0.5 <950 Kb and a jump to 0.8 at >950 Kb (Fig 4). In 16_00075, the major RDAF peak was between 0.5 and 0.6 throughout (Fig 4). 15_02015’s RDAF peaks were at 0.25 and 0.5 <220 Kb, then 0.5 at 220-950 Kb, before 0.75 at >950 Kb (Fig 4). 220 Kb and 950 Kb are at TSSs containing acH3 marks, potentially matching TSSs inferred from experiments in L. major. These results demonstrate the high level of recombination at discrete breakpoints on this chromosome*”.

9- Line 380: The RDAF in Fig3 fits with chr36 diploidy for 16_00964 and 14_01223 but not for the others. How do the authors reconcile this discrepancy? The RDAF for chr36 in 07_00242 is indeed along the 0.6 line, suggesting putative triploidy. This is even clearer for 14_00642 with RDAF signals linning close to the 0.6 and 0.3 until position ~1800 and then with only the RDAF signal at 0.3 remaining. This kind of signals are highy suggestive of triploidy. For 16_00075 the RDAF data suggests diploidy until position ~1250, and then tetraploidy from ~1250 to ~1800 with RDAF signals at 0.75 and 0.25.
The authors explain this for 16_00075 at line 410 by saying this is likely the result of 2 populations. This is possible, but not if cells were cloned prior to sequencing. This is something that should be mentionned in the Methods, i.e. were cells cloned prior to genomic DNA extraction or was genomic DNA extracted from the population. Also for how long were the cells passaged prior to genomic DNA extraction. It is mentionned in the Methods that 'isolates were grown for the minimum time necessary to produce adequate parasite numbers to isolate enough genomic DNA'. However the authors need to be more precise as passaging could have an influence here, for example by altering ploidy is cells were passaged for a long time (ploidy is known to randomly vary with time, at least for some chromosomes).

=> Close examination of Figure S6, shows that the chromosome 36 patterns for those samples are most consistent with disomy. 07_00242 has a clear mode at 0.5, and though further hypotheses on this samples are possible given the wide variations in coverage across the chromosome, our clear pattern from the coverage is that they are disomic - see the coverage across their chromosome 36 copies here (<https://drive.google.com/file/d/13RwVIN9xhyHTJYelrWNrBJ8FVpa0W8fl/view?usp=sharing>). We think 14_00642 is trisomic in the text already “*except for trisomy in 14_00642 (Fig S4)*”.

10- Line 517: It would be interesting that in the discussion the author discuss on the ploidy of chr23 in their samples (mostly diploid) compared to other Leishmania samples or species (Ltrop or others). For example in Franssen et al eLife 2020, chr23 in Ldonovani isolates is the chromosome with the highest standard deviation in ploidy value, with most isolates being triploid. Why does this uniform diploidy would be specific to Ltropica?

=> Yes, the reviewer makes a good point. For the concluding sentence of that paragraph, we now state “ *This contrasted sharply with previous work on the L. donovani complex where chromosome 23 was typically trisomic with extensive diversity and encoded an amplification of the H-locus drug-resistance region (Franssen et al 2020)*.”

11- Line 530: With next generation sequencing, it is not uncommon to observed putative amplification signal at sub-telomeric ends that are in fact artefactual. This would need to be validated, by Southern blots of digested genomic DNA followed by hybridization with a probe for a gene part of the amplicon and for a gene outside of the amplicon, to compare hybridization signal intensities (normalised by the hybridization signal for a gene not part of chr10).

=> We adjusted the phrasing on chromosome 10 to be say it *“could*” be amplified: “*The sub-telomeric regions of this chromosome could be amplified …*”. This is justified because we did not observe such extensive sub-telomeric amplifications in other samples for this chromosome, nor so substantially on other chromosomes. We also examined this in more detail with IGV (Figure S11) and showed a typical other sample (15_02480 in Figure S12).

12- Lines 531-532: This only supports triploidy is the authors sequenced clones, hence the need adding this info to the Methods, as mentionned above. If they sequenced the population of parasites isolated from the lesion one cannot exclude that this is due to a mixed population, as suggested at line 410 for chr 36.

=> We modified that section allow for this consideration as follows: “*consistent with trisomy or possibly a mixed cell population*”.

Reviewer #4: In this manuscript, a whole genome sequence strategy has been used to map SNPs in 22 Leishmania tropica isolates from treated patients in Sweden with different outcomes .These patients were infected in the Middle East in a decade (2007-2017). According to this analysis, blocks of heterozygosity followed by homozygous blocks with breakpoints at strand-switch regions have been found. The authors conclude that these findings sugggest the existence of genetic exchange in possible parasexual processes, but this requires further experimental testing. In fact, chromosome amplification as a gene expression regulation mechanism, and recombination events through mobile genetic elements, may also explain the results.Therefore, experiments to determine which mechanisms are involved in the variations found should be conducted. No experiments have been performed to suggests the existence of meiotic processes in Leishmania.
Language editing. The manuscript is well written but full of imprecise or not standard terms that should be defined. For example, homozygous differentiation, homozygous similarity or mixed heterozygosity.

=> We agree that further experimental testing/experiments are required to better understand and determine the mechanisms around genetic exchange in *Leishmania*. Other processes than parasexual recombination/mechanisms like mobile genetic elements and gene expression regulation mechanisms, have been addressed in prior comments above.

To address the reviewer’s input on the phrases “homozygous differentiation, homozygous similarity or mixed heterozygosity”, we use the long run of homozygosity (LROH) term instead. Accordingly, we modified the text to say “*The chromosome 36 of 14_01223 displayed heterozygosity at <1.63 Mb, then a LROH at 1.63-1.78 Mb, followed by near homozygosity >1.80 Mb*”. And later to: “where the pattern of heterozygosity changed to a LROH until 1.8 Mb”. And the Figure 3 legend to say “*This pattern of heterozygosity (blue), a LROH (grey) and homozygous SNPs (orange)*”. And for chromosome 2: “*followed by a LROH from this point to the end of the chromosome at 1,520 Kb (Fig 5)*”.

--------------------

PLOS authors have the option to publish the peer review history of their article (<a href="<https://eur01.safelinks.protection.outlook.com/?url=https%3A%2F%2Fjournals.plos.org%2Fplosntds%2Fs%2Feditorial-and-peer-review-process%23loc-peer-review-history&amp;data=04%7C01%7Chedvig.glans%40ki.se%7Ce50546771ce84257d03808d993148221%7Cbff7eef1cf4b4f32be3da1dda043c05d%7C0%7C0%7C637702538570508965%7CUnknown%7CTWFpbGZsb3d8eyJWIjoiMC4wLjAwMDAiLCJQIjoiV2luMzIiLCJBTiI6Ik1haWwiLCJXVCI6Mn0%3D%7C1000&amp;sdata=Vfvx8%2FJQhmtgUIHItjcPEK8QFAucAHL6h7UGASVolW4%3D&amp;reserved=0>" target="_blank">what does this mean?</a>). If published, this will include your full peer review and any attached files.<br><br>

If you choose “no”, your identity will remain anonymous but your review may still be made public.<br><br>

<b>Do you want your identity to be public for this peer review?</b> For information about this choice, including consent withdrawal, please see our <a href="<https://eur01.safelinks.protection.outlook.com/?url=https%3A%2F%2Fwww.plos.org%2Fprivacy-policy&amp;data=04%7C01%7Chedvig.glans%40ki.se%7Ce50546771ce84257d03808d993148221%7Cbff7eef1cf4b4f32be3da1dda043c05d%7C0%7C0%7C637702538570508965%7CUnknown%7CTWFpbGZsb3d8eyJWIjoiMC4wLjAwMDAiLCJQIjoiV2luMzIiLCJBTiI6Ik1haWwiLCJXVCI6Mn0%3D%7C1000&amp;sdata=UbDYiWp8UVhgCgRWO4nUzgyFLoPS%2FJQMdcCGicxS%2Bk8%3D&amp;reserved=0>" target="_blank">Privacy Policy</a>.

Reviewer #1: No

Reviewer #2: No

Reviewer #3: No

Reviewer #4: No


Figure Files:
While revising your submission, please upload your figure files to the Preflight Analysis and Conversion Engine (PACE) digital diagnostic tool, <https://eur01.safelinks.protection.outlook.com/?url=https%3A%2F%2Fpacev2.apexcovantage.com%2F&amp;data=04%7C01%7Chedvig.glans%40ki.se%7Ce50546771ce84257d03808d993148221%7Cbff7eef1cf4b4f32be3da1dda043c05d%7C0%7C0%7C637702538570508965%7CUnknown%7CTWFpbGZsb3d8eyJWIjoiMC4wLjAwMDAiLCJQIjoiV2luMzIiLCJBTiI6Ik1haWwiLCJXVCI6Mn0%3D%7C1000&amp;sdata=Q%2FrRnW15oE5M9Yx5pYxsh3SuhH8SZKc1JXZOIkD8jHs%3D&amp;reserved=0>. PACE helps ensure that figures meet PLOS requirements. To use PACE, you must first register as a user. Then, login and navigate to the UPLOAD tab, where you will find detailed instructions on how to use the tool. If you encounter any issues or have any questions when using PACE, please email us at figures@plos.org.
 
Data Requirements:
Please note that, as a condition of publication, PLOS' data policy requires that you make available all data used to draw the conclusions outlined in your manuscript. Data must be deposited in an appropriate repository, included within the body of the manuscript, or uploaded as supporting information. This includes all numerical values that were used to generate graphs, histograms etc.. For an example see here: <https://eur01.safelinks.protection.outlook.com/?url=http%3A%2F%2Fwww.plosbiology.org%2Farticle%2Finfo%253Adoi%252F10.1371%252Fjournal.pbio.1001908%23s5&amp;data=04%7C01%7Chedvig.glans%40ki.se%7Ce50546771ce84257d03808d993148221%7Cbff7eef1cf4b4f32be3da1dda043c05d%7C0%7C0%7C637702538570508965%7CUnknown%7CTWFpbGZsb3d8eyJWIjoiMC4wLjAwMDAiLCJQIjoiV2luMzIiLCJBTiI6Ik1haWwiLCJXVCI6Mn0%3D%7C1000&amp;sdata=HFORddsFyMPGgWKrf9KFlOW5ym9laKg27nnDCk3BP60%3D&amp;reserved=0>.
 
Reproducibility:
To enhance the reproducibility of your results, we recommend that you deposit your laboratory protocols in protocols.io, where a protocol can be assigned its own identifier (DOI) such that it can be cited independently in the future. Additionally, PLOS ONE offers an option to publish peer-reviewed clinical study protocols. Read more information on sharing protocols at <https://eur01.safelinks.protection.outlook.com/?url=https%3A%2F%2Fplos.org%2Fprotocols%3Futm_medium%3Deditorial-email%26utm_source%3Dauthorletters%26utm_campaign%3Dprotocols&amp;data=04%7C01%7Chedvig.glans%40ki.se%7Ce50546771ce84257d03808d993148221%7Cbff7eef1cf4b4f32be3da1dda043c05d%7C0%7C0%7C637702538570518959%7CUnknown%7CTWFpbGZsb3d8eyJWIjoiMC4wLjAwMDAiLCJQIjoiV2luMzIiLCJBTiI6Ik1haWwiLCJXVCI6Mn0%3D%7C1000&amp;sdata=gHYWW8xTg0fHUDBb%2FJhHxJrBDuW92UGJDKQWximx9jg%3D&amp;reserved=0>

__________________________________________________
In compliance with data protection regulations, you may request that we remove your personal registration details at any time.  (Use the following URL: <https://eur01.safelinks.protection.outlook.com/?url=https%3A%2F%2Fwww.editorialmanager.com%2Fpntd%2Flogin.asp%3Fa%3Dr&amp;data=04%7C01%7Chedvig.glans%40ki.se%7Ce50546771ce84257d03808d993148221%7Cbff7eef1cf4b4f32be3da1dda043c05d%7C0%7C0%7C637702538570518959%7CUnknown%7CTWFpbGZsb3d8eyJWIjoiMC4wLjAwMDAiLCJQIjoiV2luMzIiLCJBTiI6Ik1haWwiLCJXVCI6Mn0%3D%7C1000&amp;sdata=RCbyGN8ODdcA9wEJtHZ4rJPZgWy8jGGNfM5w7R1%2BdGk%3D&amp;reserved=0)>. Please contact the publication office if you have any questions.
