# Supplementary material for: High genome plasticity and frequent genetic exchange in Leishmania tropica isolates from Afghanistan, Iran and Syria
Source: PLoS Negl Trop Dis. 2021 Dec 30;15(12):e0010110. doi: 10.1371/journal.pntd.0010110 (PMC8754299; doi:10.1371/journal.pntd.0010110)
Supplement: Attachment — Submitted filename: Point-by-point responses 211206.docx [file pntd.0010110.s031.docx]

Dear Editors*,*

Thank you for your interest in our manuscript entitled “*High genome plasticity and frequent genetic exchange in Leishmania tropica isolates from Afghanistan, Iran and Syria*” (submission ID PNTD-D-21-01154, PNTD-D-21-01154R1).

We would also like to extend our thanks to the reviewers who once again provided valuable input and constructive criticism, which we have addressed point-by-point both below and in the revised version of the manuscript. The respective sections that have been modified in the revised version of the manuscript have been marked accordingly.

With these clarifications and corrections, it is our hope that the revised manuscript will be suitable for publication in *PLOS Neglected Tropical Diseases*

Yours sincerely,

Hedvig Glans, on behalf of the authors

**Point-by-point responses to the reviewers’ comments:**

The authors sequenced 22 L. tropica isolates from migrant patients originating from Syria, Iran and Afghanistan with the objective to study genetic diversity of these parasites at the isolate levels and between different isolates.
The study highlights genomic diversity in these parasites and infers genetic mechanisms underlying the observed diversity. The study does not bring major advances but confirms previous observations obtained with other strains and brings precious resources to the scientific community. As such the links to zenodo should be included to the text in addition to the link to figshare.

=> We have added a sentence in the “Data Availability” section. A link to the figshare is already in the same section.

” The sequences are available through ENA (SRA accession PRJEB45563) and Zenodo (https://doi.org/10.5281/zenodo.5645033, https://doi.org/10.5281/zenodo.5647430).”

The authors should also consider the comments about mobile genetic elements.

==> We have addressed the comment, see below.

Reviewer's Responses to Questions

<b>Key Review Criteria Required for Acceptance?</b></br></br>
As you describe the new analyses required for acceptance, please consider the following:</br></br>

<b>Methods</b></br></br>
-Are the objectives of the study clearly articulated with a clear testable hypothesis stated?</br>
-Is the study design appropriate to address the stated objectives?</br>
-Is the population clearly described and appropriate for the hypothesis being tested?</br>
-Is the sample size sufficient to ensure adequate power to address the hypothesis being tested?</br>
-Were correct statistical analysis used to support conclusions?</br>
-Are there concerns about ethical or regulatory requirements being met?</br></br>

Reviewer #1: yes

Reviewer #3: (No Response)

Reviewer #4: I am asking about the approval document from the ethical board, not the patients' consent.

==> The Ethical approval, approved by the Central Ethical Review Board in Stockholm in attached in the re-submission of the article.

--------------------

<b>Results</b></br></br>
-Does the analysis presented match the analysis plan?</br>
-Are the results clearly and completely presented?</br>
-Are the figures (Tables, Images) of sufficient quality for clarity?</br></br>

Reviewer #1: yes

Reviewer #3: (No Response)

Reviewer #4: This has been adequately addressed.

--------------------

<b>Conclusions</b></br></br>
-Are the conclusions supported by the data presented?</br>
-Are the limitations of analysis clearly described?</br>
-Do the authors discuss how these data can be helpful to advance our understanding of the topic under study?</br>
-Is public health relevance addressed?</br></br>

Reviewer #1: yes

Reviewer #3: (No Response)

Reviewer #4: The authors' haven't addressed the following issues:

"...all patients included in the study to obtain the isolates were treated with different outcomes (cure, relapse or failure). Each clinical situation and patient-parasite interaction could have resulted in different amplification patterns or recombination processes under the selective pressure. The reduced number of samples makes addressing these issues difficult. "

==> We agree, the complexity and few isolates make it difficult to correlate any links between treatment, outcome and genetic variations.

"At this preliminary stage, the results do not reach sufficient public health relevance".

==> By better understanding the genetic variations between and within different *Leishmania* and the genetic mechanisms behind the variations in combination with an increase understanding regarding the reproduction of the parasite, the more we, as a research community, will/may understand the mechanisms behind drug resistance. Gaining more information in the genome field will increase the knowledge to better treatment and maybe new drugs and molecular susceptibility tests in the future, which till improve the public health relevance.

Even when the authors have toned down the conclusions, they are merely based in suggestions. Although the topic is important, I find that the work is unfinished and the manuscript inconclusive.

This highlights the importance of properly designing experiments before conducting them.

It would be convenient to include a reference for the new sentence about the mobile genetic elements.

==> A sentence regarding mobile genetic elements with reference had been added in the re-submitted version. Line 653-654. “Recombination involving mobile genetic elements have also been suggested as a possible mechanism for the creation of mosaic aneuploidy (90).”

As the authors state in the response to the reviewers document, mobile elements are much less abundant in Leishmania spp. than in T. cruzi, but there is an exception: L. braziliensis and other species included in the Viannia subgenus.

==> We agree with the reviewer regarding the Viannia subgenus having a larger amount of retrotransposons compared to other Leishmania species. Leishmania tropica does have a relatively low mobile element content. As mentioned above, the possible involvement of mobile elements has nevertheless been mentioned in the manuscript and a reference has been included, and we agree that it should be mentioned as a possible mechanism.

<b>Editorial and Data Presentation Modifications?</b></br><br/>
Use  this section for editorial suggestions as well as relatively minor modifications of existing data that would enhance clarity. If the only modifications needed are minor and/or editorial, you may wish to recommend “Minor Revision” or “Accept”.

Reviewer #1: no comment

Reviewer #3: (No Response)

Reviewer #4: This has been adequately addressed.

--------------------

<b>Summary and General Comments</b></br></br>
Use  this section to provide overall comments, discuss strengths/weaknesses of the study, novelty, significance, general execution and scholarship. You may also include additional comments for the author, including concerns about dual publication, research ethics, or publication ethics. If requesting major revision, please articulate the new experiments that are needed.

Reviewer #1: The revised manuscript has adequately addressed my main concerns and those of the other reviewers, except for rev #4.  I am satisfied that while the paper does not provide a major advance, it does provide a valuable resource to the Leishmania population genetics community. While further studies as requested by rev#4 would indeed be required to support the mode of genetic exchange in these protists, I believe that they are beyond the scope of this paper.

Reviewer #3: (No Response)

Reviewer #4: The work is unfinished and the manuscript inconclusive (see my comments concerning the conclusions).
